# Dynamic chiral quenching of europium and terbium excited states

**DOI:** 10.1039/d5cp04880j

**Published:** 2026-03-16

**Authors:** Xinyi Wen, Dominic J. Black, Mark A. Fox, Robert Pal, David Parker

**Affiliations:** a Department of Chemistry, Hong Kong Baptist University Kowloon Tong Hong Kong China davidparker@hkbu.edu.hk; b Department of Chemistry, Durham University South Road Durham DH1 3LE UK

## Abstract

The nature of dynamic quenching of the europium or terbum excited states for both *Δ* and *Λ* stereoisomeric cationic complexes by *S* and *R* Trolox has been studied by total emission and CPL spectroscopy and with the aid of DFT computations. In parallel, investigations of the stereoselectivity of complex association with the model protein, human serum albumin have been undertaken providing an example where binding to the chiral protein leads to preferred population of a twisted square-antiprismatic conformer for the *Δ* isomer only. Non-linear Stern–Volmer quenching kinetics were observed and the stereoselective behaviour in Trolox binding was interpreted in terms of an exciplex model, in which the binding constant for exciplex formation, *K*_ex_, and the rate constant for exciplex decay, *k*_3_, determine overall quenching efficiency. Dynamic quenching was most efficient with *R* Trolox for the *Δ* Eu and Tb complexes in three different examples. Ground state DFT calculations revealed that the *Δ* charge transfer complex with *S* Trolox was 10 kJ mol^−1^ lower in energy than with *R* Trolox in the square antiprismatic conformer. However, the opposite energy order was found for the less stable twisted-square-antiprismatic conformer, suggesting that the origins of observed chiral quenching behaviour are not associated with relative free energies of each exciplex, but with their relative rates of decay.

## Abstract 2

Chiral quenching of the Tb and Eu excited state occurs preferentially for the *Δ* enantiomer by *R*-Trolox, *via* exciplex formation.

## Graphical abstract

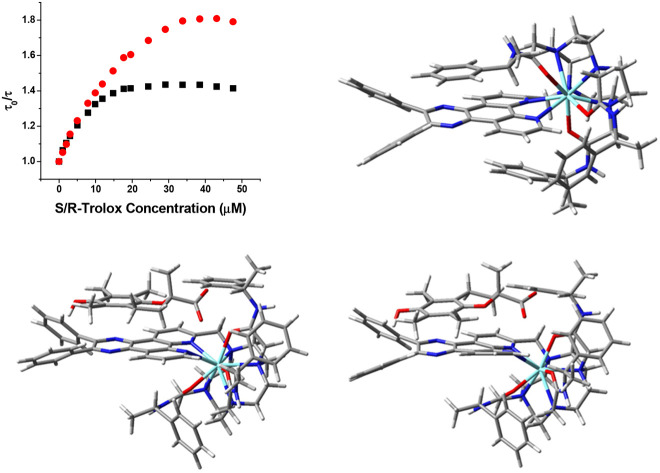

## 1. Introduction

Compelling cases of chiral recognition in the bimolecular quenching of the excited state of an enantiopure acceptor by electron transfer from a chiral donor have been difficult to define unequivocally.^1^ For singlet excited states, the typical nanosecond excited state lifetimes mean that diffusion dynamics play a key role, and many early cases exhibited a strong solvent polarity effect, suggesting that competitive aggregation may be complicating the issue.^2–5^ Here, we present evidence for chiral recognition in dynamic quenching of the long-lived metal-based excited state of *Δ* and *Λ* chiral lanthanide complexes by the enantiomers of a conformationally rigid chiral donor.

Dynamic chiral quenching has been the source of controversy with fluorescent systems for decades.^2–4,6,7^ There have been several reports of stereoselectivity in intermolecular electron transfer with systems in which a ground state donor/acceptor complex is formed.^8,9^ However, in the recent work of Vauthey and Lacour using time-resolved emission and transient absorption spectroscopy, it was noted that there was *no* experimental evidence for chiral differentiation in the excited state quenching dynamics of bimolecular systems involving photoinduced electron transfer. Their systems involved a cationic hexahelicene acceptor and stereocentred or axially chiral donors, like tryptophan or binapththol.^1^ Intuitively, remote electron transfer is not expected to show chiral differentiation; diffusion is not normally sensitive to chirality. On the other hand, if the donor has a longer-lived excited state, the acceptor will be able to undergo many diffusional encounters with it during the donor lifetime and can find the lowest energy and preferred relative orientation in the diastereoisomeric exciplexes.

In the rapid diffusion limit, charge transfer quenching of a long-lived donor in solution can be assumed to follow pseudo-first order kinetics, eqn (1)–(4), where *τ*_0_ and *τ* are emission lifetimes in the absence and presence of quencher.

1 1/*τ*_0_ = *k*_0_

2 1/*τ* = *k*_obs_

3 *k*_obs_ = *k*_0_ + *k*_2_[Q]

4 *τ*_0_/*τ* = 1 + *k*_2_/*k*_0_[Q] = 1 + *K*_SV_[Q]

When this model is valid, a plot of *τ*_0_/*τ vs.* [Q] is linear with a slope equal to *k*_2_/*k*_0_. If dynamic excited state quenching dominates over static quenching, the measured emission lifetime is assumed to be directly proportional to the emission intensity, and *I*_0_/*I* values can be used to allow estimates to be made of the Stern–Volmer quenching constant, typically expressed as *K*_SV_^−1^, and quoted in µM units.^10,11^

**Scheme 1:**
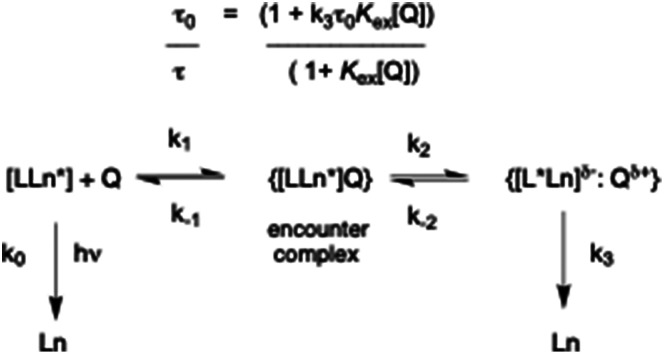
Kinetic scheme for exciplex formation.

This simple model assumes reversible formation of an encounter complex under diffusion control, followed by electron transfer between the quencher and the acceptor. Several reports have described quenching of organic chromophores by an alternative scheme, particularly for aromatic donor–acceptor pairs, in which a relatively long-lived exciplex is formed instead of direct radical–ion pair formation.^12,13^ In the alternative model (Scheme 1), an equilibrium constant can be introduced that is associated with reversible exciplex formation, in which *K*_ex_ = *k*_1_*k*_2_/*k*_−1_*k*_−2_, provided that *k*_−2_ ≫ *k*_3_.

In the alternate model, the exciplex lifetime is given by (*k*_3_)^−1^, and the ‘apparent’ Stern–Volmer constant has a rather different meaning. The measured emission lifetime of the lanthanide species, *τ*, may then vary with [Q] in a non-linear manner, and the quenching profile is determined by the relative magnitude of *K*_ex_ and the value of *k*_3_ compared to *k*_0_, the rate constant for radiative deactivation of the excited state. For example, if *K*_ex_ is of the order of 10^5^ M^−1^, and assuming a forward rate constant for association of the quencher and emissive species of 10^8^ M^−1^ s^−1^, (*i.e.* close to the diffusion limit of 10^9^/10^10^ M^−1^ s^−1^) the value of *k*_3_ is 10^3^ s^−1^ which is the same order of magnitude as the radiative rate constant for terbium or europium emission, *k*_0_. One would therefore hypothesise that the degree of quenching may become greater as the exciplex lifetime gets shorter.

In the refined version presented here (Scheme 1), the excited state energy may migrate from the lanthanide ion (Ln*) back and forth to the chromophore triplet (L*). Such a reversible energy transfer process with rates in the range 10^2^ to 10^4^ s^−1^ is thermally activated, rendering the triplet state longer-lived. This process was demonstrated recently, using picosecond and nanosecond transient absorption spectroscopy, for Eu and Tb coordination complexes with either ICT or ligand centred triplet excited states that lie above the energies of the emissive ^5^D_0_/^5^D_1_ and ^5^D_4_ states of Eu (17 200/19 100 cm^−1^) and Tb (20 400 cm^−1^), respectively.^14,15^

**Fig. 1:**
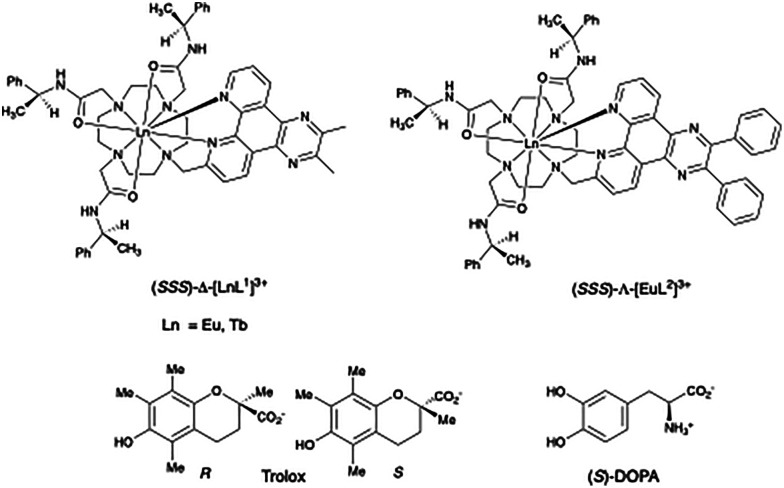
Structures of the lanthanide complexes [LnL^1,2^]^3+^ and the chiral quenching species, Trolox and DOPA.

With these rates in mind, we reasoned that it was timely to examine such longer-lived chiral Eu and Tb systems in the search for irrefutable cases of chiral recognition in excited state quenching. Therefore, we set out to study stereoselectivity in the quenching of the *Δ* and *Λ* isomers of the cationic Eu and Tb complexes of L^1^ (ref. 10 and 16–18) and in [EuL^2^]^3+^, by both the *R* and *S* isomers of the electron-rich and conformationally rigid vitamin E fragment, Trolox (Fig. 1).^19^ Examples of enantioselective quenching involving chiral lanthanide complexes are uncommon,^20,21^ and have been dominated by studies examining racemic [Ln(DPA)_3_]^3−^ complexes (DPA is the tridentate ligand, dipicolinic acid), a nine-coordinate complex that racemises quickly on the laboratory timescale. Examples of stereoselective electronic energy transfer to the quenching species were noted, although in some cases changes to the constitution of the primary emissive species, under the reaction conditions, may have been overlooked.^18^

## 2. Methods

The lanthanide(iii) complexes based on the 1,4,7,10-tetraazacyclododecane (cyclen) ligand, [LnL^1,2^]^3+^, incorporating the dipyridoquinoxaline (dpq) sensitiser, were prepared by reported methods.^15,16^ Details of solution NMR experiments and the use of emission and CPL spectroscopy to monitor dynamic quenching are given in the SI.

Yttrium(iii) is a rare earth metal ion that can be used as a diamagnetic surrogate for the central lanthanide(iii) ions. Many conformers of geometries of (*SSS*)-*Δ*-[Y**L**^2^]^3+^, (*SSS*)-*Δ*-[Y**L**^2^·*S*-Trolox]^2+^ and (*SSS*)-*Δ*-[Y**L**^2^-*R*-Trolox]^2+^ were fully optimised without symmetry constraints using the hybrid-DFT B3LYP functional^22,23^ and 3-21G* basis set for all atoms with the Gaussian 16 package.^24^ Frequency calculations confirmed all geometries to be true minima. The Gaussian16 default polarisation continuum solvent model (IEFPCM)^25^ was applied to these initial calculations with water as the solvent. The Grimme dispersion model (GD3BJ)^26^ was employed in every case to simulate correctly weak intra- and inter-molecular interactions. B3LYP/3-21G* has been shown elsewhere^27^ to be an appropriate functional/basis set method for diamagnetic Y^3+^ complexes. Further details are given in the SI.

## 3. Results and discussion

**Fig. 2:**
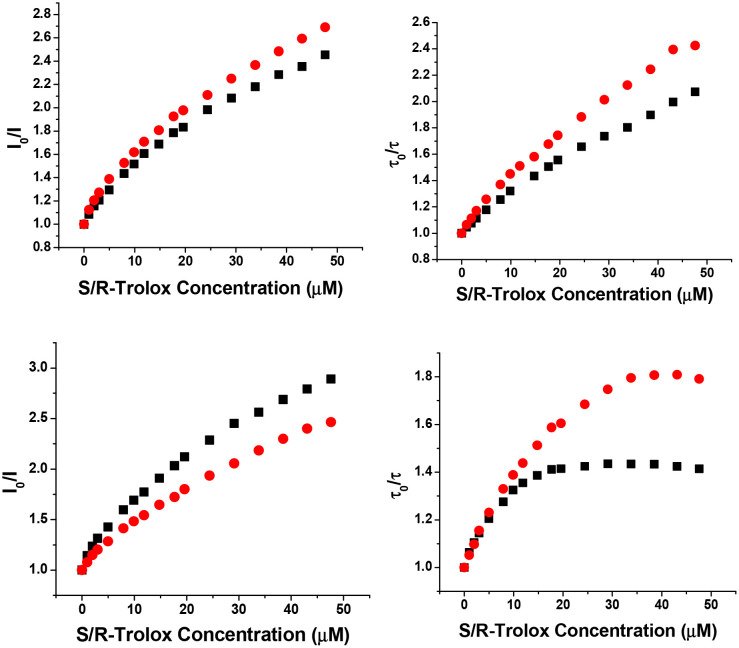
(Lower) Variation in emission intensity (left) and lifetime (right) for *Λ*-[EuL^2^]^3+^ and (upper) for *Δ*-[EuL^2^]^3+^ with *S*-Trolox (black square) and *R*-Trolox (red circle), (5 µM, pH 7.4, 50 mM HEPES, 50 mM NaCl, 296 K, *λ*_exc_ 364 nm).

**Fig. 3:**
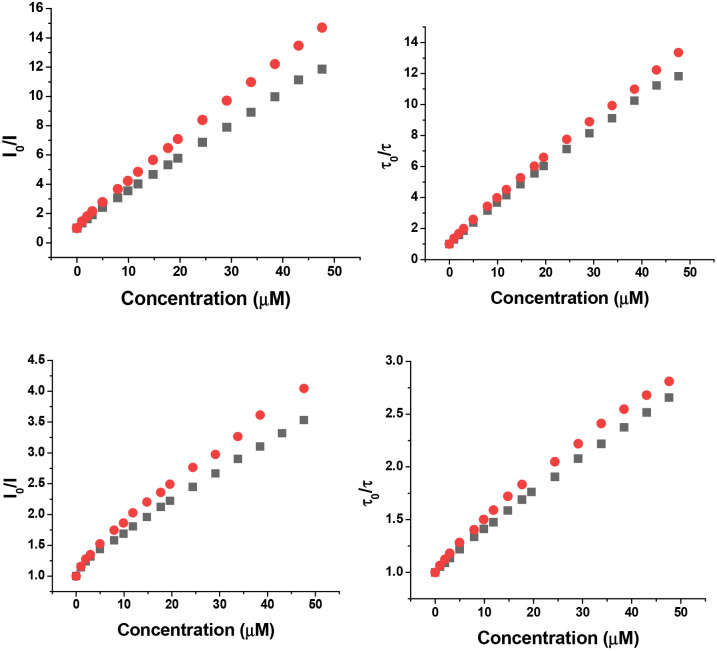
(Upper) Variation of the emission intensity (left) and lifetime (right) for *Δ*-[TbL^1^]^3+^ with *R*-Trolox (red) and *S*-Trolox (black); (lower) for *Δ*-[EuL^1^]^3+^ (5 µM, pH 7.4, 50 mM HEPES, 50 mM NaCl, 296K, *λ*_exc_ 347 nm).

Variations in the emission intensity and lifetime of the Eu and Tb complexes were examined systematically in the presence of each enantiomer of Trolox, (Table 1 and Fig. 2, 3) and in some cases by the structurally related and electron rich zwitterion, *S*-DOPA. The *Δ* Eu and Tb complexes of L^1^ and L^2^ were quenched more efficiently by *R* Trolox, and the *Λ* isomers by *S*-Trolox, as judged by lifetime variations. With *S*-DOPA, the *Λ* isomer was quenched more than the *Δ* (Fig. S1). Each Stern–Volmer plot showed deviations from linearity that are usually explained by combinations of static and dynamic quenching. The *K*_SV_^−1^ values reported in Table 1 are the concentrations at which the intensity or lifetime falls to 50% of its original value. Measurements were made in triplicate and the errors show the standard deviation values to the mean value. With the complexes of L^2^ in particular, the *Δ-R* and *Λ-S* stereoisomers showed the greatest degree of quenching, but did not show identical behaviour, as expected if behaving as enantiomeric species.

**Table 1:** Stern–Volmer quenching constant (*K*_SV_^−1^/µM ± 5%) for chloride salts of the Tb and Eu(iii) complexes of L^1^ and L^2^ by Trolox (5 µM complex, pH 7.4, 0.1 M HEPES, 298 K, *λ*_exc_ 345 or 364 nm, respectively)

Complex	*S*-Trolox (µM)		*R*-Trolox (µM)	
	Intensity/lifetime		Intensity/lifetime	
*Δ*-[EuL^1^]^3+^	16(0.6)	27(0.1)	12(0.6)	22(0.6)
*Λ*-[EuL^1^]^3+^	14(0.6)	27(0.3)	17(0.4)	29(0.2)
*Δ*-[TbL^1^]^3+^	3.4(0.4)	3.6(0.1)	2.5(0.2)	3.0(0.1)
*Λ*-[TbL^1^]^3+^	3.1(0.2)	3.3(0.1)	3.4(0.1)	3.4(0.1)
*Δ*-[EuL^2^]^3+^	26(0.3)	44(1.4)	22(0.6)	29(0.7)
*Λ*-[EuL^2^]^3+^	17(0.5)	(5)^a^	27(0.9)	(10)^a^

a Pronounced non-linearity was observed (see Fig. 2); data shown are the concentrations of quencher found to reduce *I*_0_ or *τ*_0_ by 50%. Each value represents the mean of three independent determinations with the standard deviation in brackets.

The complexes of L^1^ were more sensitive to quenching than those of L^2^, and the Tb complexes of L^1^ were most sensitive of all, reflecting in part the higher energy of the Tb ^5^D_4_ emissive excited state. No significant differences were found in emission lifetimes between aerated and degassed solutions suggesting that charge transfer dominates quenching kinetics and triplet oxygen quenching is not competitive.

The profile for *Λ*-[EuL^2^]/*S*-Trolox quenching was particularly striking, (Fig. 2, lower right) as the variation of lifetime with Trolox concentration resembled a simple binding curve for 1 : 1 association. Indeed, fitting the data to that model gave a log *K* value of 6.09 (±05), in accord with reversible exciplex formation (Fig. S2). Plugging in this estimate of *K*_ex_ into the kinetic model (Scheme 1), and using experimental values for *τ*_0_ and *τ*_0_/*τ*, when [Trolox] = 10 µM then *k*_3_ = 1.4 × 10^3^ s^−1^. With *K*_ex_ values that are one and two orders of magnitude smaller, the value of *k*_3_ increases to 1.8 × 10^3^ and 5.4 × 10^3^ s^−1^, respectively. The more stable the exciplex, the smaller *k*_3_ and the longer the exciplex lifetime.

### 3.1 ^1^H NMR studies in water and DFT computations

In the ^1^H NMR spectrum of (*SSS*)-*Δ*-[Y**L**^2^]^3+^in D_2_O, one set of phenyl ring resonances was shifted to lower frequency, with the *ortho* and *meta* protons resonating about 1 ppm to lower frequency, as observed by 1-D and 2-D COSY spectroscopy (Fig. S3). Incremental addition of *S* or *R* Trolox caused no shift of the dpq and phenyl ring resonances in the ^1^H NMR spectrum, up to 1 : 1 stoichiometry. Similarly, no change in the absorption spectrum of the dpq chromophore was evident when Trolox was added to the Y or Eu complex. Thus, addition of Trolox does not perturb the ground state and electronic structures of the complex (Fig. S4).

**Fig. 4:**
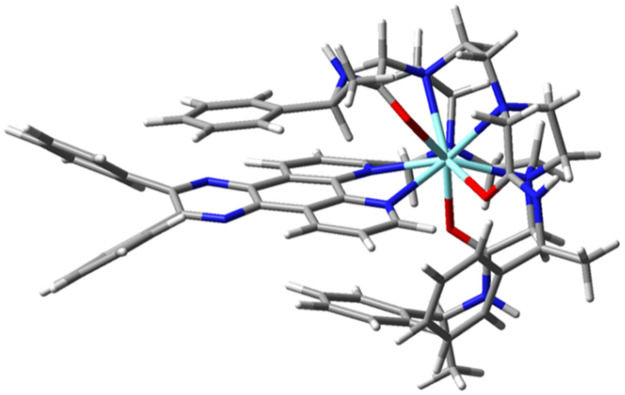
Molecular geometry of (*SSS*)-*Δ*-[YL^2^]^3+^ optimised in Gaussian16 using DFT with B3LYP/3-21G* model chemistry, showing the upper phenyl ring close in space to the pyrazine ring of the chromophore (see SI and Tables S1–S3 for further details). Yttrium is chosen as a surrogate for Eu in these calculations.

Molecular geometries of the chiral isomers of the yttrium complexes (*SSS*)-*Δ*-[YL^2^]^3+^ and (*RRR*)-*Λ*-[YL^2^]^3+^ were calculated using hybrid density functional theory (DFT), in which the lowest energy form showed that one of the pendant arms adjacent to the chromophore is located directly above the aromatic portion of the chromophore (Fig. 4), giving rise to the deshielding effect observed in the solution ^1^H NMR spectra of [YL^2^]^3+^ isomers (Fig. 1 and Fig. S3). Other located minima for [YL^2^]^3+^, but with higher energies, included different orientations for the phenyl groups of the chromophore, as well as examples with *cis*- instead of *trans*-amide units.

**Fig. 5:**
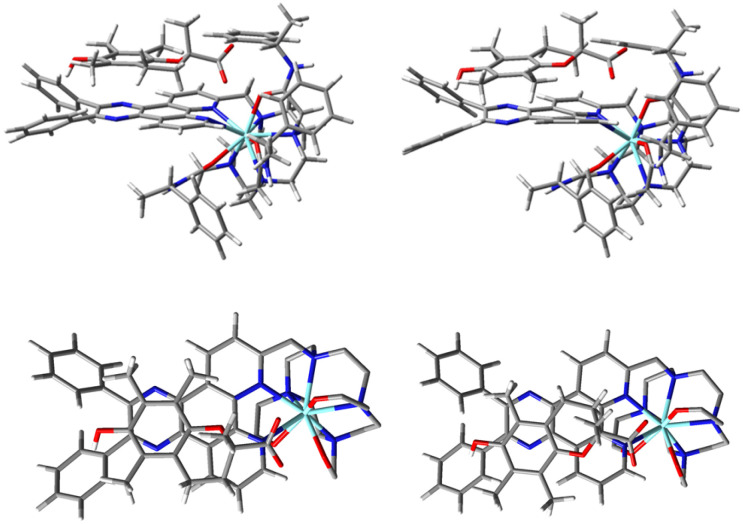
(Upper) Molecular geometries of (*SSS*)-*Δ*-[Y**L**^2^-*S*-Trolox]^2+^ (left) and (*SSS*)-*Δ*-[Y**L**^2^-*R*-Trolox]^2+^ (right); (lower): views showing the slightly different π–π overlap between the chromophore pyrazine ring and the electron rich aryl ring, with shortest distances of 3.18 and 3.21 Å respectively.

The molecular geometries of the diastereoisomeric complexes (*SSS*)-*Δ*-[YL^2^-*S*-Trolox]^2+^ and (*SSS*)-*Δ*-[YL^2^-*R*-Trolox]^2+^ derived from association of the tri-cation (*SSS*)-*Δ*-[YL^2^]^3+^ and the anion Trolox were also determined by hybrid DFT (Fig. 5). (*SSS*)-*Δ*-[YL^2^-*S*-Trolox]^2+^ was found to be lower in energy by 10 kJ mol^−1^ compared to (*SSS*)-*Δ*-[YL^2^-*R*-Trolox]^2+^. With the (*RRR*)-*Λ*-[YL^2^] series, mirror image behaviour was found for the alternate pair of diastereoisomers with *R* or *S* Trolox, *i.e.* the (*RRR*)-*Λ*-[YL^2^] complex with *R* Trolox was lower in energy by 10 kJ mol^−1^. The carboxylate group at the stereogenic carbon in the Trolox adduct is directed towards the cationic metal centre, and the geminal methyl group then lies away from the bulky ligand amide substituents. In each of these structures, intramolecular phenyl ring participation was not present, and if it was enforced it gave structures of much higher energy than those shown in Fig. 5. Such a finding suggests that approach of Trolox to the electron poor chromophore displaces the intramolecular phenyl π–π interaction.

These systems have been modelled by DFT in their ground states, but experimentally the different quenching values of the four diastereomeric pairs of europium complex [EuL^2^]^3+^ and Trolox relate to the complex excited states (*i.e.* the tetra-azatriphenylene triplet state and the lanthanide excited state) which are not explored by DFT, owing to the computational limitations associated with modelling such open-shell, paramagnetic systems.^28^

The conformers of the two chiral isomers [YL^2^]^3+^ with the two phenyl groups of the chromophore at different orientations are only 3.7 kJ mol^−1^ higher in energy than the lowest energy geometries (Fig. S5). The energy barrier associated with phenyl rotation between two conformers of a chiral isomer was estimated to be 18.8 kJ mol^−1^.

One hypothesis considered to explain why the four isomer combinations of the europium complex [LnL^1,2^]^3+^ (Ln = Eu or Tb) with Trolox have different experimental quenching values is that the stereoisomers of [EuL^1,2^]^3+^ could have different phenyl group orientations. However, this situation seems unlikely, given the low energy barrier to phenyl rotation in the axially chiral biaryl. Furthermore, the L^1^ series of complexes, with methyl groups instead of phenyl ring substituents in the chromophore, shows the same overall trend of stereoselective quenching behaviour.

### 3.2 Stereoselectivity in protein binding to human serum albumin: regulation of complex conformation

The binding of the Tb and Eu complexes to human serum albumin was studied to assess stereoselectivity in protein association. The study was carrried out because it had earlier been found that human serum albumin binding with related complexes favoured complexation of the *Δ* enantiomer, and was associated with a conformational change of the chiral complex from a square-antiprismatic (SAP) to a twisted square antiprismatic (TSAP) conformation.^16–18,29–33^

**Fig. 6:**
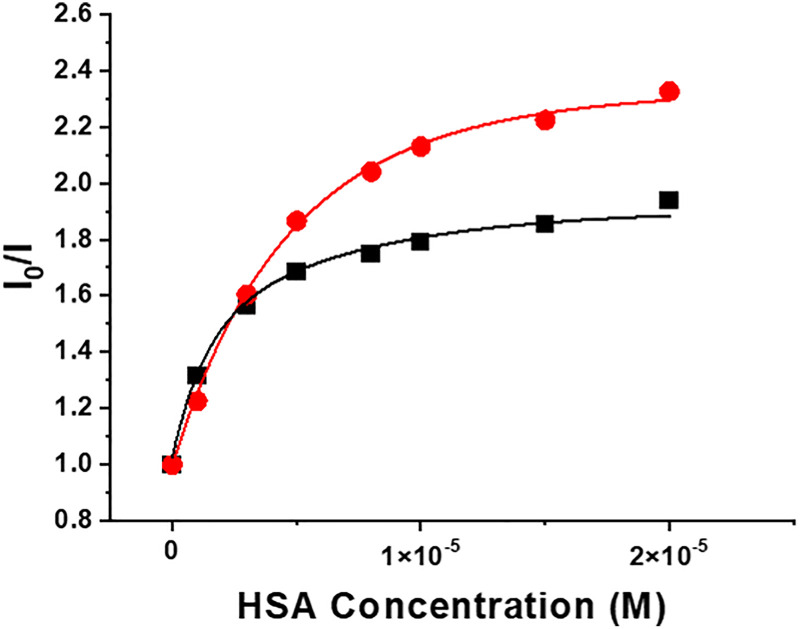
Variation of europium emission intensity at 620 nm for (*SSS*)-*Δ*-[EuL^2^]^3+^ (black) and (*RRR*)-*Λ*-[EuL^2^]^3+^(red) following addition of human serum albumin (pH 7.4 10 mM HEPES, 5 µM complex, 298 K). The line shows the fit to a 1 : 1 binding model giving log *K* values of 5.10 (05) and 5.65(07), respectively, as the mean of three independent measurements.

The changes in emission intensity were monitored as a function of added protein concentration, assuming a dominant 1 : 1 binding stoichiometry, as suggested by a multitude of related studies with HSA which binds with highest affinity to drug site 1 with these sorts of complex.^29^ Experimental values were fitted to this model, using non-linear, least-squares iterative analysis (Fig. 6, Table 2 and Fig. S6–S8). With complexes of L^1^, the *Δ* enantiomers bound more strongly, and the Tb complexes gave higher binding constants compared to their Eu analogues. However, with [EuL^2^]^3+^, the trend was reversed, without any apparent change in the absorption or emission spectral fingerprints of the free and protein-bound forms.

**Table 2:** Protein association constants (log *K*) for the chloride salts of Tb and Eu(iii) complexes of L^1^ and L^2^ (5 µM complex, pH 7.4, 10 mM HEPES, 50 mM NaCl, 296 K, *λ*_exc_ 345 or 364 nm). Values represent the mean for three independent measurements with the standard deviation in parenthesis

Complex	log *K*
*Δ*-[EuL^1^]^3+^	5.25 (02)
*Λ*-[EuL^1^]^3+^	4.67 (02)
*Δ*-[TbL^1^]^3+^	5.47 (06)
*Λ*-[TbL^1^]^3+^	4.72 (02)
*Δ*-[EuL^2^]^3+^	5.10 (05)
*Λ*-[EuL^2^]^3+^	5.65 (05)

### 3.3 Circularly polarised luminescence spectroscopy studies

CPL spectroscopy intrisically affords greater spectral resolution than total emission spectroscopy, as both the sign and relative intensities of the observed transitions can be readily distinguished.^30^

**Fig. 7:**
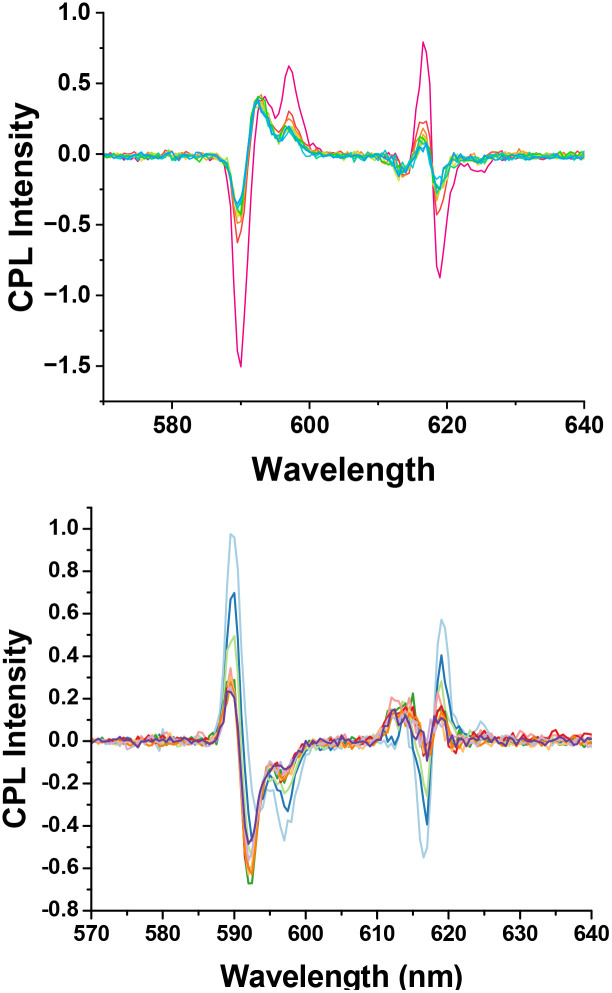
(Upper) CPL spectra for (*RRR*)-*Λ*-[EuL^2^]^3+^ following addition of HSA (red = zero to green = 100 µM) showing quenching of each emission band; (lower): for (*SSS*)-*Δ*-[EuL^2^]^3+^ (light blue – zero to dark blue 100 µM HSA); pH 7.4, 50 mM HEPES, 50 mM NaCl, 296 K, *Λ*_exc_ 365 nm, revealing the changes in relative intensity and small shifts in energy of the CPL transitions observed around 595 and 615 nm.

With [EuL^2^]^3+^, protein binding to the *Λ* isomer simply gave rise to reductions in the intensity of the Δ*J* = 1 and 2 manifolds, without any change in energy (Fig. 7). In contrast, the *Δ*-enantiomer showed changes in the separation and relative intensity of the three transitions in the magnetic-dipole allowed series around 590–600 nm. Indeed, the energy separation of the major components of Δ*J* = 1 manifold was reduced in the protein-bound form, consistent with a reduction in the second-order crystal field coefficient, a parameter that is used to assess ligand field strength in lanthanide coordination complexes.^31,32^ Similarly, distinctive changes were also evident in the five electric dipole-allowed transitions of the Δ*J* = 2 manifold.

**Fig. 8:**
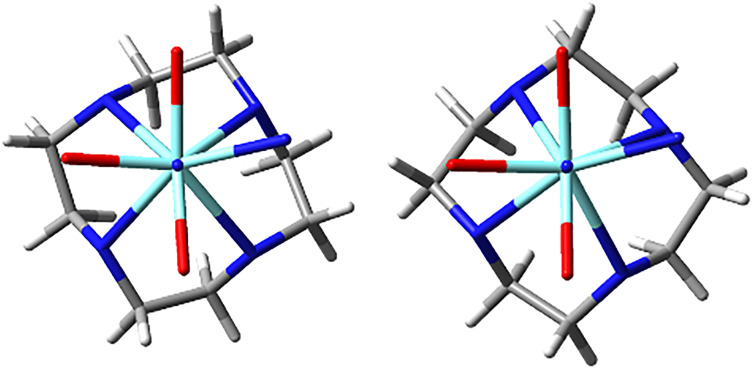
Simplified SAP (left) and TSAP (right) geometries of (*SSS*)-*Δ*-[YL^2^]^3+^ viewed along the *N*–*Y* axis, showing the tetraazacyclododecane macrocycle and the atoms coordinated to the *Y* ion.

Taken together, such behaviour can be interpreted in terms of reversible switching of *Δ*-[EuL^2^]^3+^, from a square-antiprismatic *Δ*-(λλλλ) coordination geometry, to a twisted square antiprismatic polyhedron with a *Δ*-(δδδδ) configuration in its HSA complex; this conformational exchange process involves cooperative ring inversion of the 12-N_4_ macrocycle, a process that is decoupled from isomer interconversion *via* arm rotation^33,34^ (Fig. 8). Dynamic exchange of these isomers has been reported earlier^33^ with Tb analogues, leading to dynamic helicity inversion. Cooperative ring inversion in such complexes occurs in solution at a rate of 50 s^−1^ at ambient temperature and typically is associated with a free energy of activation of about 60 kJ mol^−1^.^35^ Inspection of the Eu emission spectral fingerprints of *Δ*-[EuL^1^]^3+^ in free and protein bound forms reveal similar changes, again for the *Δ* enantiomer only (Fig. S8).

The ground state dynamic exchange process, interconverting SAP and TSAP isomers observed in protein binding, may also occur for these complexes in their excited states, during exciplex formation with *R* or *S* Trolox. That being so, then the different sensitivities to quenching of the four stereoisomeric excited state complexes (*Δ*/*Λ* with *R*/*S* Trolox: Table 1) can be rationalised by the differing reactivities of the SAP and TSAP isomeric complexes with *R* or *S* Trolox. The quenched species is not observable by emission or CPL spectroscopy, unlike the situation observed in HSA association, where the protein-bound form remains emissive (Fig. 7).

Accordingly, additional DFT calculations were undertaken to examine the relative stability of the *Δ* and *Λ* [YL^2^]^3+^ complexes with *R* or *S* Trolox, in both SAP and TSAP coordination geometries. The TSAP geometry for the isolated [YL^2^]^3+^ complex (Fig. S9) was found to be 20.3 kJ mol^−1^ higher in energy than the SAP geometry. With Trolox present, the TSAP geometry of (*SSS*)-*Δ*-[YL^2^-*S*-Trolox]^2+^ was higher in energy by 29.9 kJ mol^−1^ compared to (*SSS*)-*Δ*-[YL^2^-*R*-Trolox]^2+^. In contrast, the energy difference between the TSAP and SAP (*SSS*)-*Δ*-[YL^2^-*R*-Trolox]^2+^ geometries was only 10.0 kJ mol^−1^ (Table 3).

**Table 3:** Calculated relative energies in kJ mol^−1^ of the two lowest energy square antiprism (SAP) conformers of (*SSS*)-*Δ*-[YL^2^-*S*-Trolox]^2+^ and (*SSS*)-*Δ*-[YL^2^-*R*-Trolox]^2+^ and their twisted square antiprism (TSAP) analogues: see also Tables S1–S3. The same free energies occur for the mirror image isomers

Complex	Conformer	Electronic energy kJ mol^−1^	Gibbs free energy kJ mol^−1^
(*SSS*)-*Δ*-(λλλλ)-[YL^2^-*S*-Trolox]^2+^	SAP	0	0
(*SSS*)-*Δ*-(λλλλ)-[YL^2^-*R*-Trolox]^2+^	SAP	14.2	10.0
(*SSS*)-*Δ*-(δδδδ)-[YL^2^-*S*-Trolox]^2+^	TSAP	34.9	29.9
(*SSS*)-*Δ*-(δδδδ)-[YL^2^-*R*-Trolox]^2+^	TSAP	30.6	20.0

As found with the more thermodynamically stable SAP complexes (Fig. 5 and Table 3), the Trolox anion occupies the space next to the electron poor chromophore in the TSAP geometries of (*SSS*)-*Δ*-[YL^2^-*S*-Trolox]^2+^ and (*SSS*)-*Δ*-[YL^2^-*R*-Trolox]^2+^ (Fig. S10 and S11). Stabilising π–π overlap occurs between the chromophore pyrazine moiety and the electron rich aryl ring, with shortest distances of 3.26 and 3.39 Å, respectively.

In the (*SSS*)-*Δ*-[YL^2^-*R*-Trolox]^2+^ excited state system, given their relatively small energy of separation (about 4 times *kT*), the SAP and TSAP conformers can co-exist in the ground state at room temperature. It is plausible that the less thermodynamically stable TSAP complex is the more kinetically active, in accordance with the Curtin–Hammett principle. Thus, the non-identical quenching behaviour observed experimentally can be rationalised by the interplay between relative exciplex stability and the differing free energies of activation for exciplex decay. In principle, such a hypothesis could be tested in future by exploiting transient absorption spectroscopy to monitor exciplex formation and decay; such a study might be able to define more closely the origins of the unique chiral quenching behaviour reported here.

## 4. Conclusions

In the ground state structures of the cationic lanthanide complexes, [LnL^1^]^3+^ (Ln = Eu and Tb) and [EuL^2^]^3+^, ^1^H NMR and hybrid DFT studies reveal the presence of a stabilising intramolecular π–π interaction between the electron-poor dpq sensitiser and a proximate phenyl ring of the chiral amide moiety. This stabilising interaction disappears during the millisecond lifetime of the metal excited state, following approach of an electron rich Trolox anion. Instead, a stabilising π–π interaction occurs that lowers the triplet energy of the dpq excited state and enables back energy transfer to take place between the metal excited state and the dpq T_1_ state. The latter is quenched by charge transfer. The overall efficiency of quenching of the *Δ* and *Λ* stereoisomeric complexes by *R* and *S* Trolox is a complex interplay between the relative thermodynamic stabilities of stereoisomeric exciplexes, *K*_ex_ and their lifetimes, 1/*k*_3_.

The dynamic quenching behaviour of (*SSS*)-*Δ*-[EuL^2^]^3+^ with the chiral protein human serum albumin hints at a possible rationalisation for the different quenching behaviour observed with Trolox. The *Δ* complex, but not its *Λ* enantiomer, binds more weakly to HSA and undergoes a SAP to TSAP conformational change on protein binding, as deduced by CPL spectroscopy. It is reasonable to hypothesise that the occurrence of this dynamic equilibrium could also be important in the excited state chiral quenching behaviour observed with *R* and *S* Trolox. The *Δ* isomeric Eu/Tb complexes are quenched more strongly by *R* Trolox than the *Λ* isomers, in every case (Table 1).

In the more stable SAP exciplex structures, DFT calculations revealed that (*SSS*)-*Δ*-[YL^2^-*S*-Trolox]^2+^ was lower in energy by 10 kJ mol^−1^ compared to (*SSS*)-*Δ*-[YL^2^-*R*-Trolox]^2+^. However, in the TSAP geometry, (*SSS*)-*Δ*-[YL^2^-*S*-Trolox]^2+^ was higher in free energy by 30 kJ mol^−1^ compared to (*SSS*)-*Δ*-[YL^2^-*R*-Trolox]^2+^ (Table 3). Moreover, the energy difference between the TSAP and SAP (*SSS*)-*Δ*-[YL^2^-*R*-Trolox]^2+^ geometries is only 10 kJ mol^−1^.

Bringing together all this information, we hypothesise that this study provides a unique case of chiral quenching of a lanthanide excited state, where all four diastereoisomeric complexes have been examined for the first time. The different experimental quenching profiles of the stereoisomeric *Δ-SSS* and *Λ-RRR* complexes by *R* and *S*-Trolox can be explained in terms of the Curtin–Hammett principle. The *Δ* complexes populate a kinetically more reactive TSAP conformer in their exciplexes with *R*-Trolox, while the *Λ* isomers adopt the SAP geometry in their excited state complexes. With *S*-Trolox, the *Δ*-complex preferentially populates the SAP conformer, while the *Λ*-complex is more efficiently quenched when the exciplex adopts a TSAP conformer. We conclude that the chiral quenching profiles have a kinetic rather than a thermodynamic origin.

## Author contributions

The project was conceived by DP; the manuscript was written by DP and MAF; XW carried the synthesis and characterisation, measurements of binding constants, rate constants and lifetimes. DJB/RP recorded CPL spectra.

## Conflicts of interest

There are no conflicts to declare.

## Supplementary Material

CP-028-D5CP04880J-s001

## Data Availability

Any additional data, notable the Cartesian coordinates of the TSAP-DFT structures, can be obtained by request to the corresponding author. The additional experimental data associated with this article have been included in the supplementary information (SI). Supplementary information: computational methods, photophysical data. See DOI: https://doi.org/10.1039/d5cp04880j.

## References

[cit1] Varma P., Nancoz C., Bosson J., Labrador G. M., Lacour J., Vauthey E. (2023). Phys. Chem. Chem. Phys..

[cit2] Irie M., Yorozu T., Hayashi K. (1978). J. Am. Chem. Soc..

[cit3] Rau H., Totter F. (1992). J. Photochem. Photobiol., A.

[cit4] Pu L. (2004). Chem. Rev..

[cit5] Woerner M., Greiner G., Rau H. (1995). J. Phys. Chem..

[cit6] Shili Q., Yangyang S., Xudong H., Hongtao C., Lidi G., Zhongyu H., Dongsheng Z., Xinyao L., Sibing Z. (2021). RSC Adv..

[cit7] Pu L. (2020). Angew Chem., Int. Ed..

[cit8] Takashima H., Tanaka M., Hasegawa Y., Tsukahara T. (2003). J. Biol. Inorg. Chem..

[cit9] Imai Y., Kamon K., KInuta T., Tajima N., Sato T., Kuroda R., Matsubara Y. (2007). Tetrahedron.

[cit10] Rehm D., Weller A. (1969). Ber. Bunsen-Ges..

[cit11] Rehm D., Weller A. (1970). Isr. J. Chem..

[cit12] Kuzmin M. G. (1993). Pure Appl. Chem..

[cit13] Dossot,D. Burget M., Allonas X., Jacques P. (2001). New J. Chem..

[cit14] Cheung T. L., Ju Z., Zhang W., Parker D., Deng R. (2024). ACS Appl. Mater. Interfaces.

[cit15] Wen X., Li H., Ju Z. Y., Deng R., Parker D. (2024). Chem. Sci..

[cit16] Poole R. A., Bobba G., Cann M. J., Frias J.-C., Parker D., Peacock R. D. (2005). Org. Biomol. Chem..

[cit17] Montgomery C. P., New E. J., Parker D., Peacock R. D. (2008). Chem. Commun..

[cit18] Carr R., Evans N. H., Parker D. (2012). Chem. Soc. Rev..

[cit19] Cordes T., Vogelsang J., Tinnefeld P. (2009). J. Am. Chem. Soc..

[cit20] Yamashita J., Iwamura M., Takanishi T., Nozaki K., Tsubomura T. (2025). ChemistryEurope.

[cit21] Li Y. L., Wang H.-L., Zhu Z.-H., Wang Y.-F., Liang F.-P., Zou H.-H. (2024). Nat. Commun..

[cit22] Becke A. D. (1993). J. Chem. Phys..

[cit23] Lee C., Yang W., Parr R. G. (1988). Phys. Rev. B: Condens. Matter Mater. Phys..

[cit24] Frisch M. J. , Trucks G. W., Schlegel H. B., Scuseria G. E., Robb M. A., Cheeseman J. R., Scalmani G., Barone V., Petersson G. A., Nakatsuji H., Li X., Caricato M., Marenich A. V., Bloino J., Janesko B. G., Gomperts R., Mennucci B., Hratchian H. P., Ortiz J. V., Izmaylov A. F., Sonnenberg J. L., Williams-Young D., Ding F., Lipparini F., Egidi F., Goings J., Peng B., Petrone A., Henderson T., Ranasinghe D., Zakrzewski V. G., Gao J., Rega N., Zheng G., Liang W., Hada M., Ehara M., Toyota K., Fukuda R., Hasegawa J., Ishida M., Nakajima T., Honda Y., Kitao O., Nakai H., Vreven T., Throssell K., Montgomery, Jr. J. A., Peralta J. E., Ogliaro F., Bearpark M. J., Heyd J. J., Brothers E. N., Kudin K. N., Staroverov V. N., Keith T. A., Kobayashi R., Normand J., Raghavachari K., Rendell A. P., Burant J. C., Iyengar S. S., Tomasi J., Cossi M., Millam J. M., Klene M., Adamo C., Cammi R., Ochterski J. W., Martin R. L., Morokuma K., Farkas O., Foresman J. B. and Fox D. J., Gaussian 16, Revision B.01, Gaussian, Inc., Wallingford CT, 2016

[cit25] Tomasi J., Mennucci B., Cancès E. (1999). J. Mol. Struct.: THEOCHEM.

[cit26] Grimme S., Ehrlich S., Goerigk L. (2011). J. Comput. Chem..

[cit27] Neil E. R., Fox M. A., Pal R., Pålsson L.-O., O’Sullivan B. A., Parker D. (2015). Dalton Trans..

[cit28] Verma P., Truhlar D. G. (2020). Trends Chem..

[cit29] Li H., Black D. J., Han W., Ng S. L.-W., Pal R., Parker D. (2025). Chem. Sci..

[cit30] Li D., Jiang Z., Liu X., Cheng Y. (2026). Chem. Biomed. Imaging.

[cit31] Parker D., Suturina E. A., Kuprov I., Chilton N. F. (2020). Acc. Chem. Res..

[cit32] Binnemanns K. (2015). Coord. Chem. Rev..

[cit33] Parker D., Fradgley J. D., Delbianco M., Starck M., Walton J. W., Zwier J. M. (2022). Faraday Discuss..

[cit34] Parker D., Fradgley J. D., Wong K.-L. (2021). Chem. Soc. Rev..

[cit35] Parker D., Dickins R. S., Puschmann H., Crossland C., Howard J. A. K. (2002). Chem. Rev..

