# Supplementary material for: Dynamic chiral quenching of europium and terbium excited states
Source: Phys Chem Chem Phys. 2026 Mar 16;28(14):8769–75. doi: 10.1039/d5cp04880j (PMC13004223; doi:10.1039/d5cp04880j)
Supplement: CP-028-D5CP04880J-s001 [file CP-028-D5CP04880J-s001.pdf]

Supplementary Information (SI) for Physical Chemistry Chemical Physics.
This journal is © the Owner Societies 2026

***Supplementary Information***

# Dynamic chiral quenching of europium and terbium excited sates

Xinyi Wen [a], Dominic J. Black [b], Mark A. Fox [b], Robert Pal [b] and David Parker [a*†]

a) Department of Chemistry, Hong Kong Baptist University, Kowloon Tong, 999077, Hong Kong, China

b) Department of Chemistry, Durham University, South Road, Durham DH1 3LE, UK


| | |
|---|---|
| **1. General Procedures and optical techniques** | Pages 2-3 |
| **2. Quenching: $[EuL^2]^{3+}$ with *S*-DOPA and *S*-Trolox; Figs S1-2** | Pages 4 |
| **3. $^1H$ NMR Spectra of $[YL^2]Cl_3$; Figs S3-4** | Pages 5-6 |
| **4. DFT Calculation of phenyl ring rotations; Figs S5** | Pages 6 |
| **5. Protein binding behaviour for $[LnL^1]^{3+}$; Figs S6-8** | Pages 7-8 |
| **6. DFT analysis: yttrium complexes; Figs S9-11, Tables S1-3** | Pages 9-11 |
| **7. References** | Pages 11-12 |
| **8. List of Cartesian Coordinates** | Pages 13-20 |

## Experimental

### General procedures

All reagents were obtained from commercial sources and were used as received. Solvents were laboratory grade, and anhydrous solvents were obtained from a Solvent Purification System. Air sensitive and moisture sensitive reactions were conducted under an inert atmosphere of nitrogen using Schlenk-line techniques.

**$^1$H NMR spectra** were obtained using a 9.4 T Bruker Ultrashield 400 Plus NMR spectrometer ($^1$H NMR at 400 MHz; $^{13}$C NMR at 101 MHz) at 295 K. The $^1$H NMR chemical shifts were referenced to the corresponding solvent peak (4.79 for $D_2O$). Deuterated solvents were all commercially available. All chemical shifts are given in ppm with coupling constants in Hz.

### Optical techniques

**UV/Vis absorbance** spectra were recorded using an Agilent Technologies Cary 8454 UV-Vis spectrometer.

**Emission spectra** were recorded on a HORIBA Scientific Fluoromax-4 luminescence spectrofluorometer.

**Lifetime measurements** were carried out using a HORIBA Fluorolog-3 spectrometer under the stated conditions.

**Temperature dependent measurements** were carried out using Peltier-controlled cuvette holder supplied with magnetic stirring using T-App software.

**CPL spectra** were recorded with a home-built (modular) spectrometer. The excitation source was a broad band (200 – 1000 nm) laser- driven light source EQ 99 (Elliot Scientific). The excitation wavelength was selected by feeding the broadband light into an Acton SP-2155 monochromator (Princeton Instruments); the collimated light was focused into the sample cell (1 cm quartz cuvette). Sample PL emission was collected perpendicular to the excitation direction with a lens (f = 150 mm). The detection of the CPL signal was achieved using the field modulation lock-in technique. The electronic signal from the PMT was fed into a lock-in amplifier (Hinds Instruments Signaloc Model 2100). The reference signal for the lock-in detection was provided by the PEM control unit. The monchromators, PEM control unit and lock-in amplifier were interfaced to a

desktop PC and controlled by a custom-written Labview graphic user interface. Spectral calibration of the scanning monochromator was performed using a Hg-Ar calibration lamp (Ocean Optics). The emission spectra were recorded with 0.5 nm step size, and the slits of the detection monochromator were set to a slit width corresponding to a spectral resolution of 0.25 nm. CPL spectra (as well as total emission spectra) were obtained through an averaging procedure of several scans. The CPL spectra were smoothed using a shape-preserving Savitzky-Golay smoothing (polynomial order 5, window size 9 with reflection at the boundaries) to reduce the influence of noise and enhance visual appearance; all calculations were carried out using raw spectral data. Analysis of smoothed vs raw data was used to help to estimate the uncertainty in the stated gem factors, which was typically ±10%.

**Supplementary Information Figures:**

**Quenching: $[EuL^2]^{3+}$ with L-DOPA and *S*-Trolox**

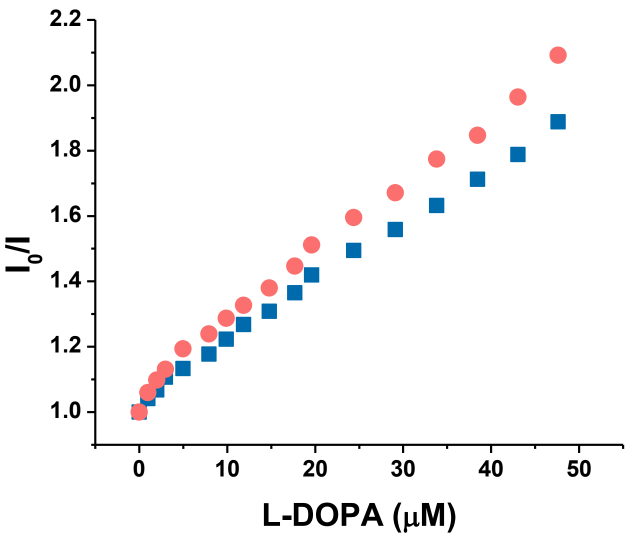


**Fig. S1** Variation of europium emission intensity at 615 nm for (*SSS*)-Δ-$[EuL^2]^{3+}$ (blue square) and (*RRR*)-Λ-$[EuL^2]^{3+}$(red circle) following addition of *S*-DOPA (pH 7.4 0.1 M HEPES, 5 μM complex, 298 K).

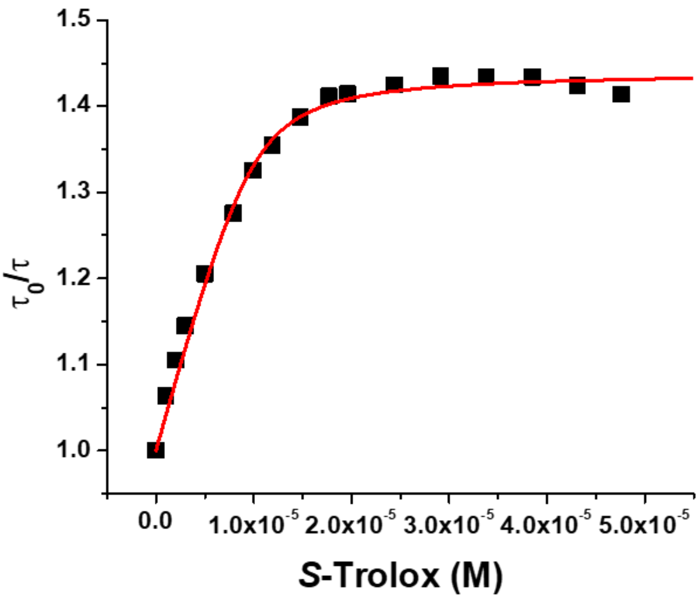


**Fig. S2** Variation of europium emission lifetime at 615 nm for (*RRR*)-Λ-$[EuL^2]^{3+}$ following addition of *S*-Trolox (pH 7.4 0.1 M HEPES, 5 μM complex, 298 K). Data fitting to a 1:1 binding model gave a mean log *K* value of 6.09 (07), from 3 independent measurements

## $^{1}H$ NMR Spectra of $[YL^2]Cl_3$

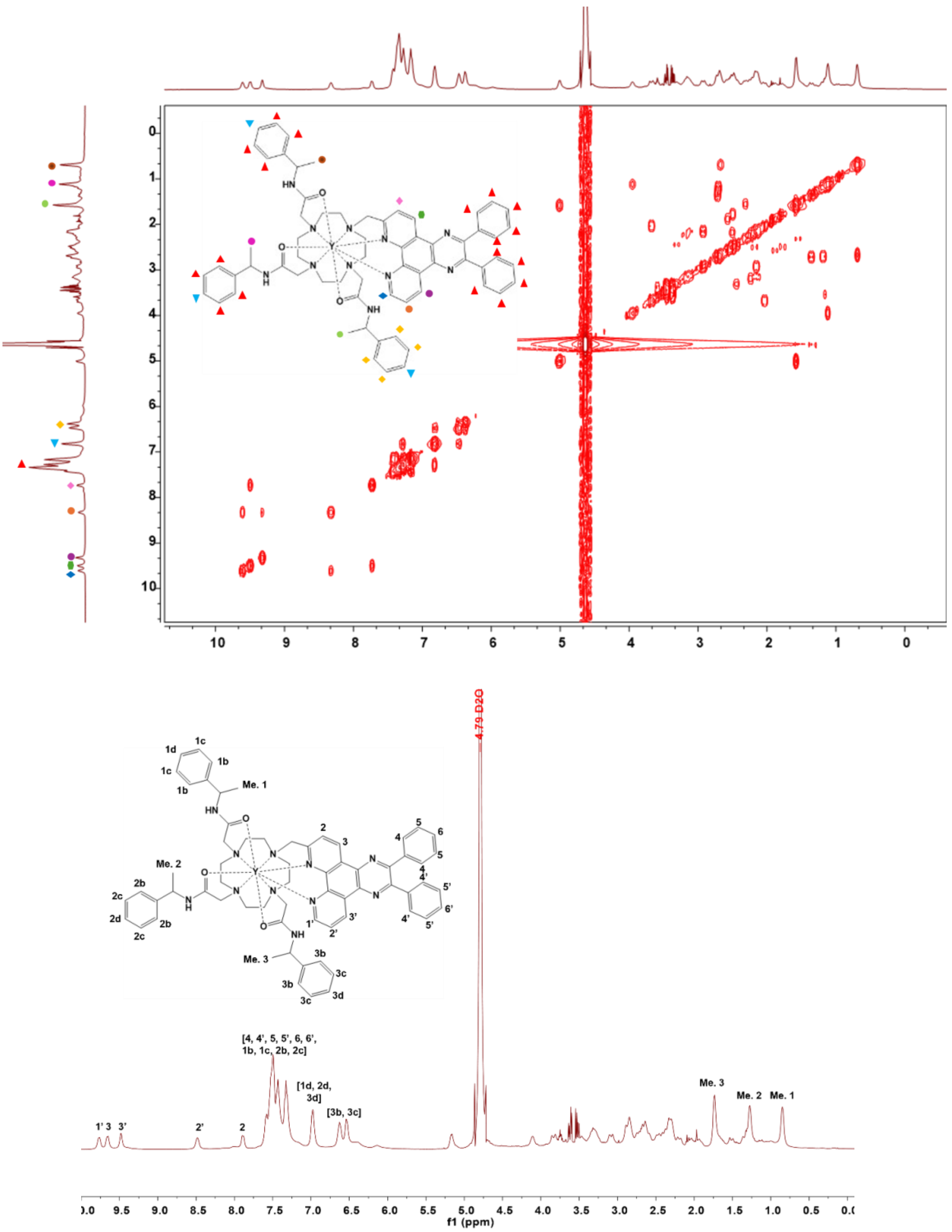


**Fig. S3** (*Upper*): $^{1}H$ NMR COSY and (*lower*) 1D spectrum spectra of (*RRR*)-Λ-$[YL^2]^{3+}$, (5 mM complex, 296 K, 9.4 T, $D_2O$) highlighting the aromatic ring proton assignments and the shifted resonances of the phenyl protons (3b, 3c) that resonate to lower frequency of other aromatic ring protons, owing to the proximity to the ring current associated with the dipyridoquinoxaline chromophore.

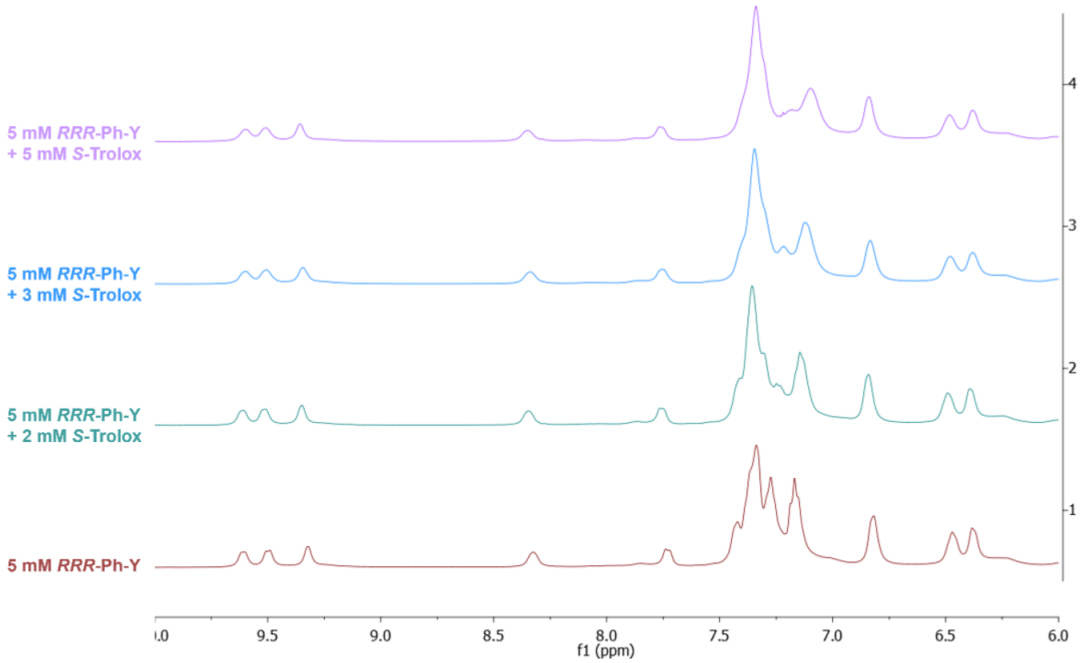


**Fig. S4** Expansion of the $^{1}H$ NMR spectrum of (*RRR*)-Λ-$[YL^2]^{3+}$, (5 mM complex, 296 K, 9.4 T, $D_2O$) highlighting the absence of change when *S*-Trolox is added to the complex.

## DFT Calculations of phenyl ring rotations

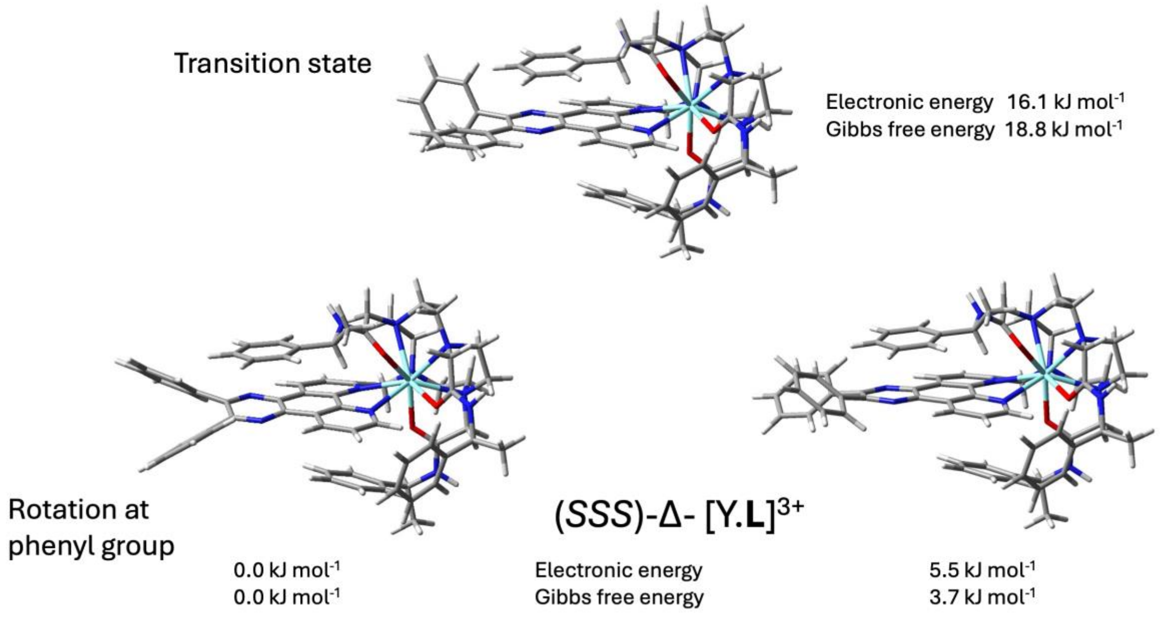


**Fig. S5** Geometries and energies of the phenyl group rotations in the chromophore of (*SSS*)-Δ-$[YL^{\mathbf{2}}]^{3+}$.

## Protein binding behaviour for $[LnL^1]^{3+}$

### (*SSS*)-Δ-$[LnL^1]^{3+}$

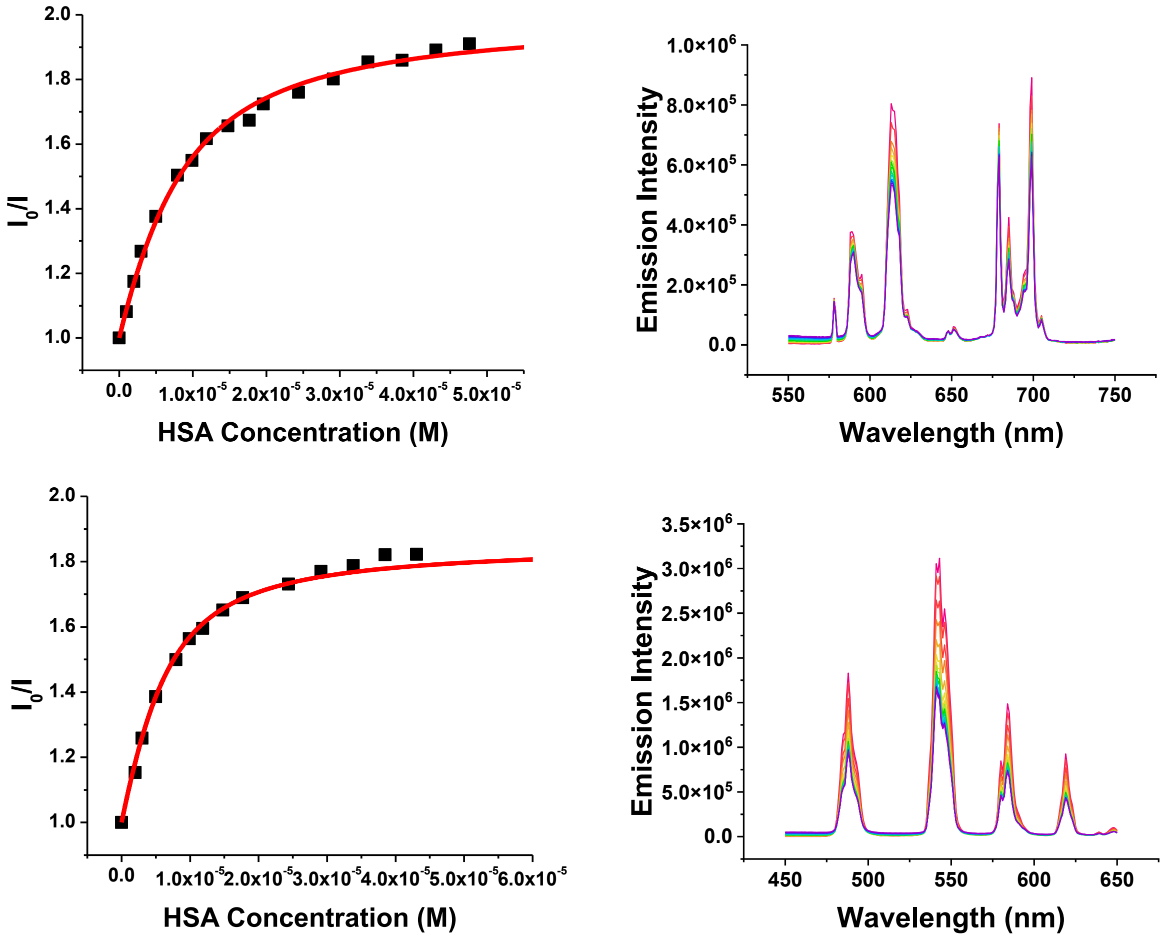


**Fig. S6** (*Upper*) Variation of lanthanide emission intensity at 613 nm for (*SSS*)-Δ-$[EuL^1]^{3+}$ and (*lower*) at 541 nm for (*SSS*)-Δ-$[TbL^1]^{3+}$, following incremental addition of human serum albumin (pH 7.4 10 mM HEPES, 5 μM complex, 298 K). Fitting to a 1:1 binding model gave mean log *K* values of 5.25 (02) and 5.47(06), respectively.

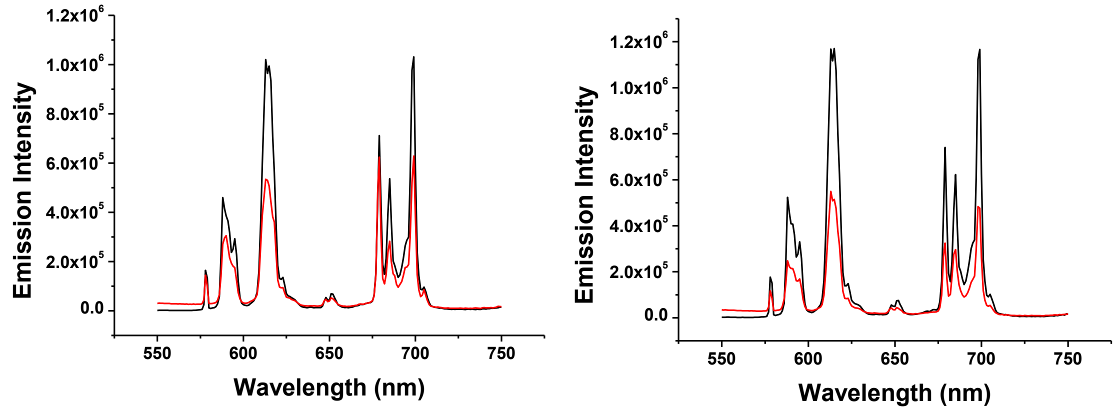


**Fig. S7** Comparison of the Eu emission spectra before (*black*) and after (*red*) HSA addition: (*left*): (*SSS*)-Δ-[EuL[1]]$^{3+}$; (*right*): (*RRR*)-Λ-[EuL[1]]$^{3+}$. Small but distinct changes in the relative intensity and energy of transitions around the Δ$J$ = 1 and Δ$J$ = 4 manifolds in particular are apparent with the Δ isomer that are not evident with the Λ isomer, where a consistent percentage reduction in intensity can be observed in each transition.

**(*RRR*)-Λ-[EuL[1]]$^{3+}$**

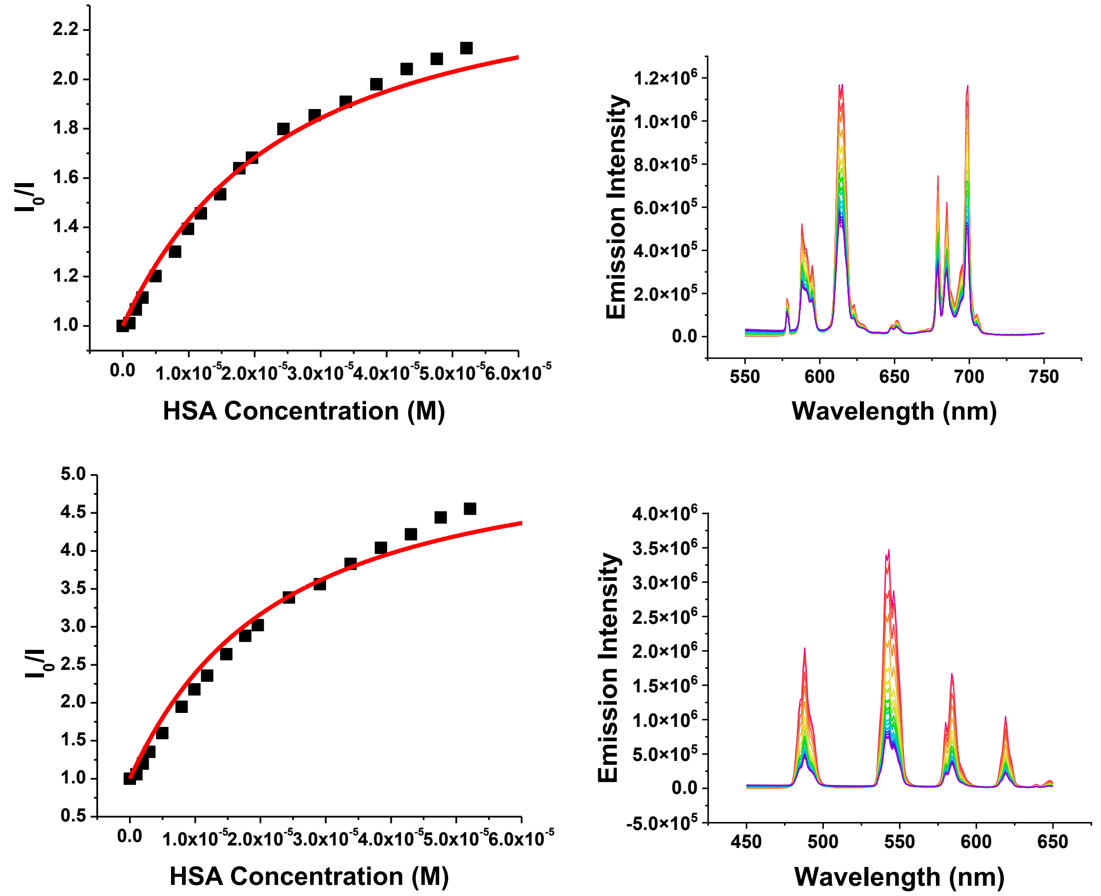


**Fig. S8** (*Upper*) Variation of lanthanide emission intensity at 613 nm for (*RRR*)-Λ-[EuL[1]]$^{3+}$ and (*lower*) at 541 nm for (*RRR*)-Λ-[TbL[1]]$^{3+}$, following incremental addition of human serum albumin (pH 7.4 10 mM HEPES, 5 μM complex, 298 K). Fitting to a 1:1 binding model gave mean log $K$ values of 4.67 (02) and 4.72(02), respectively.

### DFT analysis: yttrium complexes

Many conformers of geometries of (*SSS*)-Δ-[Y**L²**]$^{3+}$, (*SSS*)-Δ-[Y**L²**.*S*-Trolox]$^{2+}$ and (*SSS*)-Δ-[Y**L**$^2$-*R*-Trolox]$^{2+}$ were fully optimised without symmetry constraints using the hybrid-DFT B3LYP functional[1,2] and 3-21G* basis set for all atoms with the Gaussian 16 package.[3] Frequency calculations confirmed all geometries to be true minima. The Gaussian16 default polarisation continuum solvent model (IEFPCM)[4] was applied to these initial calculations with water as solvent. The Grimme dispersion model (GD3BJ) [5] was employed in every case to simulate correctly weak intra- and inter-molecular interactions. B3LYP/3-21G* has been shown elsewhere [6] to be an appropriate functional/basis set method for diamagnetic $Y^{3+}$ complexes.

The optimised geometries in Figures 4, 5 and S5 were generated using GaussView software.[7] The binding energy for each Trolox dication, (*SSS*)-Δ-[Y**L**$^2$-*S*-Trolox]$^{2+}$ or (*SSS*)-Δ-[Y**L**$^2$-*R*-Trolox]$^{2+}$ was obtained as the difference between the sum of the energies of the isolated (*SSS*)-Δ-[Y**L²**]$^{3+}$ trication and Trolox anion and the energy of the Trolox pair (Table S3). The high binding energies reflect the favourable charge attractions between the two isolated ions. The transition state geometry of the phenyl rotations of the chromophore was obtained with the OPT=TS3 command in Gaussian16.

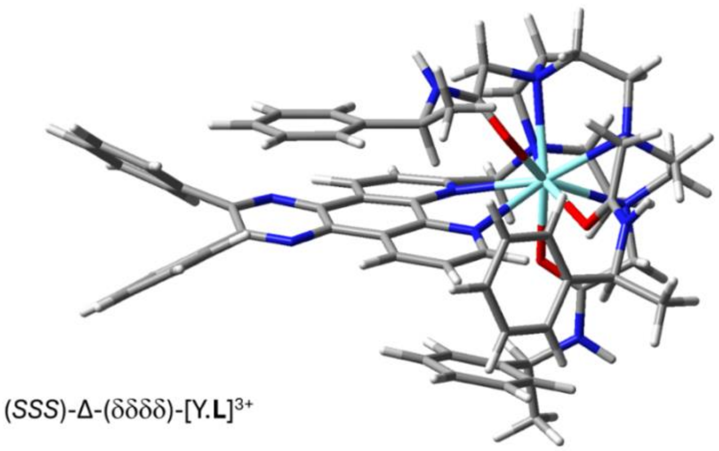


**Fig. S9** Molecular geometry of TSAP (*SSS*)-Δ-[YL$^2$]$^{3+}$.

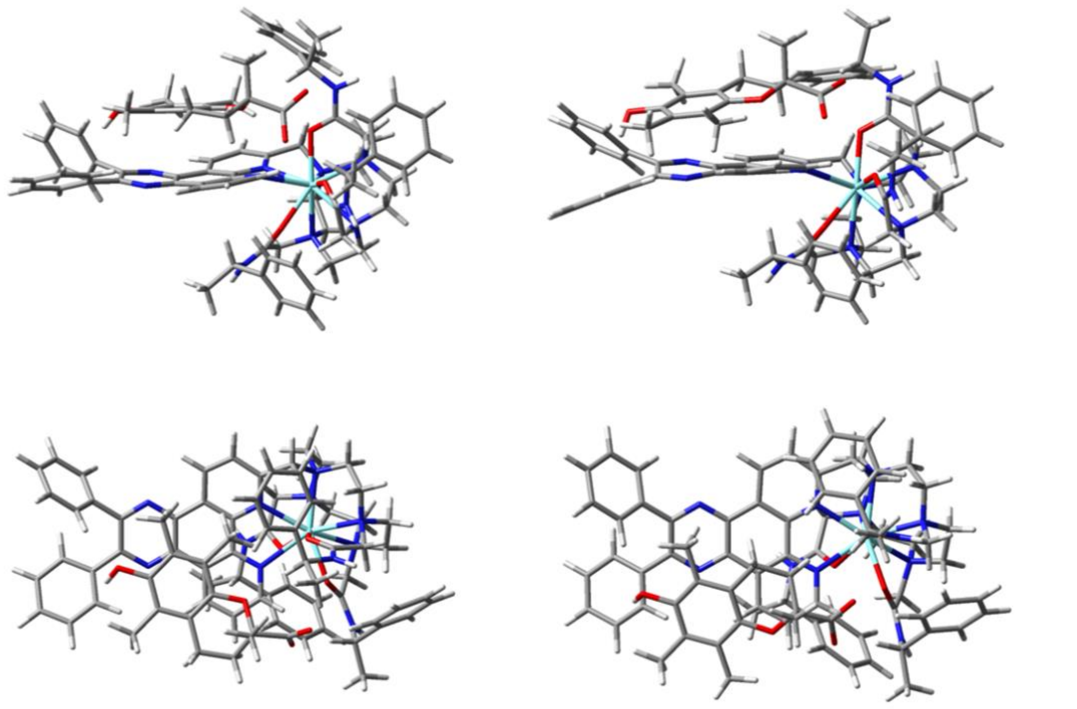

**Fig. S10** (*Upper*): Molecular TSAP geometries of (*SSS*)-Δ-(δδδδ)-[$YL^2$-*S*-Trolox]$^{2+}$ (*left*) and (*SSS*)-Δ-(δδδδ)-[$YL^2$-*R*-Trolox]$^{2+}$ (*right*); (*lower*): views showing the slightly different π-π overlap between the chromophore pyrazine ring and the electron rich aryl ring, with shortest distances of 3.26 and 3.39 Å respectively.

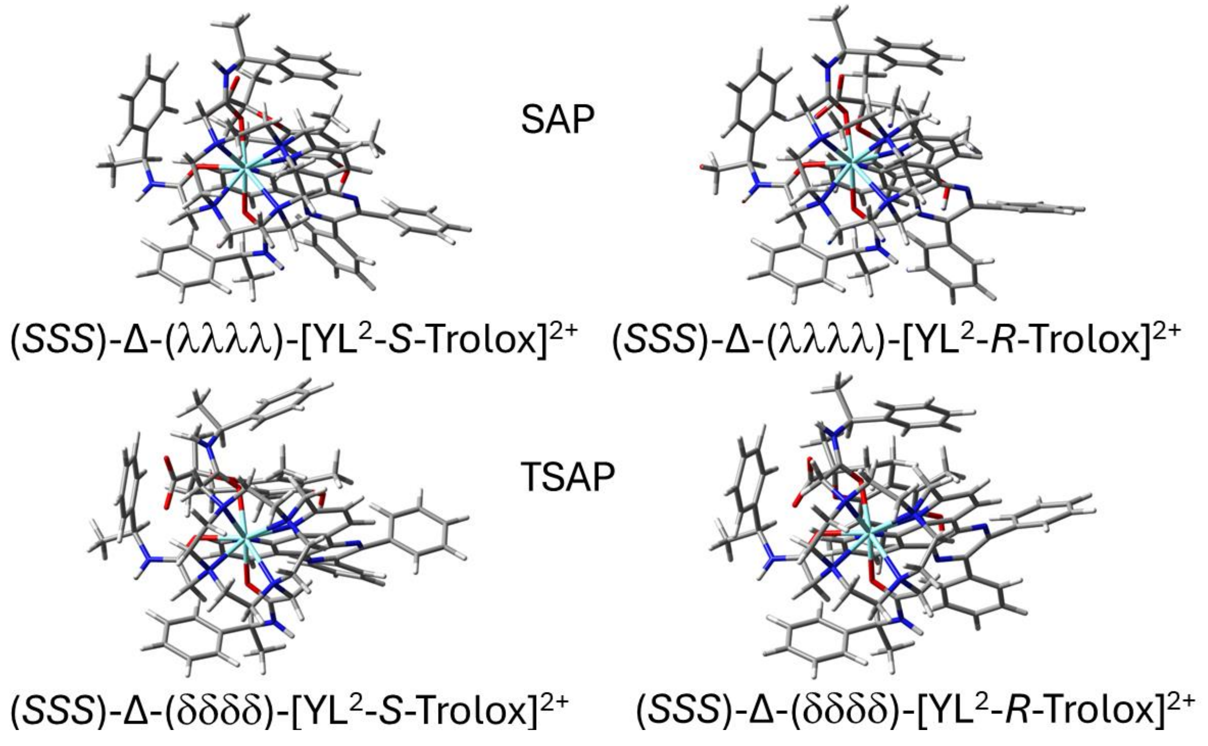


**Fig. S11** (*Left*): Molecular SAP and TSAP (*SSS*)-Δ- [$YL^2$-*S*-Trolox]$^{2+}$ geometries, (*right*): SAP and TSAP (*SSS*)-Δ- [$YL^2$-*R*-Trolox]$^{2+}$ geometries with views of different 12-$N_4$ ring conformations.

**Table S1** Relative energies in kJ mol$^{-1}$ of the two lowest energy conformers of (*SSS*)-Δ-[YL$^2$-*S*-Trolox]$^{2+}$ and (*SSS*)-Δ-[YL$^2$-*R*-Trolox]$^{2+}$.

| | Electronic | Gibbs free |
|---|---|---|
| (*SSS*)-Δ-[**YL$^2$**-*S*-Trolox]$^{2+}$ | 0 | 0 |
| (*SSS*)-Δ-[YL$^2$-*R*-Trolox]$^{2+}$ | 14.2 | 10.0 |

**Table S2** Trolox binding energies in kJ mol$^{-1}$ of (*SSS*)-Δ-[YL$^2$-*S*-Trolox]$^{2+}$ and (*SSS*)-Δ-[YL$^2$-*R*-Trolox]$^{2+}$.

| | Electronic | Gibbs free |
|---|---|---|
| (*SSS*)-Δ-[YL$^2$*S*-Trolox]$^{2+}$ | 287.3 | 192.6 |
| (*SSS*)-Δ-[YL$^2$-*R*-Trolox]$^{2+}$ | 273.1 | 182.6 |

**Table S3** Relative energies in kJ mol$^{-1}$ of (*SSS*)-Δ-[YL$^2$]$^{3+}$ with different phenyl group orientations at the chromophore.

| | Electronic | Gibbs free |
|---|---|---|
| Conformer 1 | 0.0 | 0.0 |
| Transition state | 16.1 | 18.8 |
| Conformer 2 | 5.5 | 3.7 |

**References**


1. A. D. Becke, *J. Chem. Phys.*, 1993, **98**, 5648-5652.
2. C. Lee, W. Yang and R. G. Parr, *Phys. Rev. B. Condens. Matter*, 1988, **37**, 785-789.
3. Gaussian 16, Revision B.01; M. J. Frisch, G. W. Trucks, H. B. Schlegel, G. E. Scuseria, M. A. Robb, J. R. Cheeseman, G. Scalmani, V. Barone, G. A. Petersson, H. Nakatsuji, X. Li, M. Caricato, A. V. Marenich, J. Bloino, B. G. Janesko, R. Gomperts, B. Mennucci, H. P. Hratchian, J. V. Ortiz, A. F. Izmaylov, J. L. Sonnenberg, D. Williams-Young, F. Ding, F. Lipparini, F. Egidi, J. Goings, B. Peng, A. Petrone, T. Henderson, D. Ranasinghe, V. G. Zakrzewski, J. Gao, N. Rega, G. Zheng, W. Liang, M. Hada, M. Ehara, K. Toyota, R. Fukuda, J. Hasegawa, M. Ishida, T. Nakajima, Y. Honda, O. Kitao, H. Nakai, T. Vreven, K. Throssell, J. A. Montgomery, Jr., J. E. Peralta, F. Ogliaro, M. J. Bearpark, J. J. Heyd, E. N. Brothers, K. N. Kudin, V. N. Staroverov, T. A. Keith, R. Kobayashi, J.

Normand, K. Raghavachari, A. P. Rendell, J. C. Burant, S. S. Iyengar, J. Tomasi, M. Cossi, J. M. Millam, M. Klene, C. Adamo, R. Cammi, J. W. Ochterski, R. L. Martin, K. Morokuma, O. Farkas, J. B. Foresman, and D. J. Fox, Gaussian, Inc., Wallingford CT, 2016.

4. J. Tomasi, B. Mennucci, and E. Cancès, *J. Mol. Struct. (Theochem)*, 1999, **464**, 211-226.

5. S. Grimme, S. Ehrlich and L. Goerigk, *J. Comp. Chem.*, 2011, **32**, 1456-65.

6 E. R. Neil, M. A. Fox, R. Pal, L.-O. Pålsson, B. A. O'Sullivan and D. Parker, *Dalton Trans.*, 2015, **44**, 14937–14951.

7 GaussView, Version 6; R. Dennington, T. Keith and J. Millam, Semichem Inc., Shawnee Mission KS, 2019.

## Cartesian Coordinates

(*SSS*)-Δ-[Y.L**2**]$^{3+}$
149 atoms
B3LYP/3-21G*//IEFPCM-water//GD3BJ
NIMAG=0
Electronic Energy = -6646.562174 a.u.
Gibbs Free Energy = -6645.395142 a.u.

```
O  1.264517 -0.563199 -2.177830
O  3.582815  1.008096 -0.561342
O  2.887388 -0.687138  1.894990
N  3.019723 -3.331822  0.886817
N  2.043776 -3.110907 -1.870719
N  4.091931 -0.964517 -2.383208
N  5.069665 -1.186796  0.406578
N  0.575763 -1.898645  0.748303
C  3.108332 -4.479825 -0.089867
C  1.980911 -4.413006 -1.103460
C  3.146535 -3.216172 -2.905944
C  3.562689 -1.866714 -3.475960
C  5.513964 -1.359846 -2.053874
C  5.996528 -0.788516 -0.721713
C  5.415339 -2.598287  0.832227
C  4.319750 -3.257322  1.660553
C  1.891032 -3.602236  1.852475
C  0.725384 -2.888683 -2.546985
C  0.475160 -1.405400 -2.717688
C  4.038155  0.461764 -2.857173
C  4.147829  1.367355 -1.652056
C  5.229576 -0.230037  1.559146
C  3.970115 -0.256759  2.408749
C  0.556643 -3.084804  1.381343
C -0.655107 -3.699636  1.736637
C -1.849844 -3.049123  1.467199
C -1.824369 -1.752399  0.924860
C -0.575829 -1.196660  0.598723
H  3.061733 -5.432593  0.451818
H  4.075900 -4.430021 -0.587627
H  2.039983 -5.266776 -1.788708
H  1.018628 -4.451814 -0.592632
H  2.820051 -3.872985 -3.721500
H  4.003033 -3.686315 -2.424386
H  4.331960 -2.023724 -4.241264
H  2.719074 -1.364884 -3.943124
H  6.187430 -1.031397 -2.855356
H  5.547108 -2.448458 -2.018503
H  7.010727 -1.156664 -0.526471
H  6.052137  0.296806 -0.761861
H  6.350650 -2.590688  1.405558
H  5.590033 -3.174101 -0.076664
H  4.648098 -4.264738  1.943955
H  4.137395 -2.698792  2.575304
H  2.111685 -3.037511  2.763243
H  1.838805 -4.666814  2.102431
H -0.066036 -3.280727 -1.901770
H  0.668419 -3.412987 -3.508312
H  3.043425  0.620995 -3.283683
H  4.804338  0.668798 -3.610091
H  5.312086  0.775303  1.144921
H  6.123439 -0.453952  2.150136
H -0.642654 -4.666258  2.221157
H -2.807092 -3.493627  1.703703
Y  2.617299 -1.092639 -0.320370
C -0.467579  0.180567  0.174495
C -1.628715  0.954248  0.019504
C  0.894492  1.991354 -0.322803
C -1.493187  2.294373 -0.377234
C -0.222775  2.811984 -0.544908
H  1.892214  2.385005 -0.405822
H -2.386128  2.886623 -0.517804
H -0.060118  3.841850 -0.820661
N  0.779854  0.694165  0.005142
C -2.921383  0.377038  0.316032
C -3.023446 -0.963567  0.729871
C -5.225088  0.621536  0.511693
C -5.341741 -0.783499  0.751708
N -4.020701  1.154823  0.258830
N -4.234406 -1.524516  0.922906
N  4.008606  0.184205  3.657923
N  4.816115  2.508296 -1.741761
N -0.622426 -1.037337 -3.366709
C -1.099963  0.369870 -3.402998
C  4.984192  3.402777 -0.563192
C  2.821293  0.079542  4.560214
C  3.158861  0.796485  5.878951
H  4.049499  0.360898  6.343883
H  3.329316  1.860395  5.689265
H  2.316788  0.692910  6.567145
C  6.147900  4.367158 -0.847767
H  7.066186  3.810538 -1.058392
H  5.901861  5.007572 -1.700630
H  6.307729  5.001415  0.027230
C -0.654687  1.020595 -4.725084
H  0.431164  0.929199 -4.818603
H -1.135877  0.524272 -5.572073
H -0.912366  2.081987 -4.737574
H  4.885263  0.507201  4.062268
H -1.173945 -1.731411 -3.869127
H  5.277708  2.766498 -2.611796
C -2.612765  0.387798 -3.180354
C -3.361862  1.518393 -3.532032
C -3.275863 -0.703461 -2.600610
C -4.745742  1.539159 -3.348012
H -2.872577  2.384435 -3.958066
C -4.662039 -0.689777 -2.432242
H -2.714520 -1.568858 -2.274340
C -5.402364  0.428767 -2.813573
H -5.310971  2.418608 -3.632530
```

```
H -5.157892 -1.547715 -1.995983
H -6.476917  0.444310 -2.682620
C  3.712884  4.170600 -0.244747
C  3.366222  4.394708  1.091081
C  2.936560  4.733343 -1.263938
C  2.262712  5.189792  1.409425
H  3.964450  3.953689  1.881132
C  1.834545  5.530386 -0.946648
H  3.195145  4.552348 -2.300494
C  1.496626  5.762082  0.390777
H  2.006675  5.365566  2.447300
H  1.243163  5.971962 -1.740145
H  0.642071  6.380881  0.635414
C  1.562047  0.662255  3.938066
C  1.564621  1.929347  3.349366
C  0.365903 -0.058871  4.015873
C  0.380317  2.473454  2.850184
H  2.494576  2.479316  3.261507
C -0.821798  0.489478  3.529300
H  0.367062 -1.053854  4.448494
C -0.817635  1.760806  2.950060
H  0.397600  3.443826  2.371322
H -1.743408 -0.077033  3.590738
H -1.736415  2.184576  2.565926
H  2.653767 -0.986068  4.749678
H  5.230050  2.762413  0.286901
H -0.600063  0.869595 -2.571662
C -6.623799 -1.518436  0.768635
C -6.757571 -2.639766  1.601157
C -7.671591 -1.176321 -0.101786
C -7.936756 -3.382608  1.595214
H -5.932041 -2.911918  2.245546
C -8.842899 -1.930473 -0.116379
H -7.566650 -0.323736 -0.757542
C -8.982952 -3.028937  0.737809
H -8.039146 -4.236535  2.253565
H -9.644045 -1.664212 -0.794718
H -9.898410 -3.607831  0.730614
C -6.361822  1.563404  0.546242
C -6.348568  2.668438 -0.318519
C -7.412859  1.416312  1.465536
C -7.394190  3.589539 -0.295870
H -5.522128  2.775603 -1.007019
C -8.448905  2.347355  1.494896
H -7.414695  0.579039  2.149976
C -8.448026  3.429934  0.608819
H -7.387373  4.429728 -0.979474
H -9.254542  2.231783  2.209434
H -9.260497  4.146133  0.628201
```

(*SSS*)-Δ-[Y.L$^2$*S*-Trolox]$^{2+}$

184 atoms

B3LYP/3-21G*//IEFPCM-water//GD3BJ

NIMAG=0

Electronic Energy -7486.884332 a.u.

Gibbs Free Energy -7485.438208 a.u.

```
O  1.114999  2.252608  0.987433
O  3.411092  1.182391 -0.711996
O  2.983364 -1.631366  0.197226
N  2.868792 -1.215361  3.182049
N  1.693212  1.469111  3.460432
N  3.825004  2.553570  1.621695
N  4.983161 -0.147061  1.359906
N  0.475273 -0.912927  1.736354
C  2.806953 -0.489090  4.504734
C  1.617721  0.456248  4.577034
C  2.697863  2.534652  3.854703
C  3.171185  3.397689  2.691387
C  5.236570  2.234974  2.047108
C  5.829990  1.096849  1.231720
C  5.288864 -0.828002  2.675586
C  4.231310 -1.859859  3.054929
C  1.814221 -2.296992  3.159070
C  0.346771  2.097300  3.260365
C  0.291924  2.671631  1.862307
C  3.832678  3.316806  0.328565
C  3.899128  2.357567 -0.835570
C  5.318134 -1.069688  0.225329
C  4.181952 -2.038600  0.015222
C  0.465937 -1.745788  2.788880
C -0.738174 -2.099115  3.422745
C -1.928279 -1.548337  2.969195
C -1.924599 -0.729893  1.823280
C -0.693688 -0.484383  1.197411
H  2.747634 -1.218924  5.321998
H  3.736083  0.066668  4.624551
H  1.602686  0.957152  5.552806
H  0.686593 -0.096307  4.468049
H  2.258891  3.178437  4.627655
H  3.550720  2.024235  4.300091
H  3.884066  4.138896  3.074011
H  2.348877  3.943189  2.237226
H  5.867226  3.127574  1.948347
H  5.210919  1.961920  3.102110
H  6.853241  0.897263  1.572681
H  5.881998  1.358653  0.175408
H  6.269455 -1.316624  2.615976
H  5.350251 -0.055596  3.441658
```

| | | | |
|---|---|---|---|
| H | 4.516604 | -2.331935 | 4.003473 |
| H | 4.162933 | -2.646468 | 2.306339 |
| H | 2.093439 | -2.994086 | 2.368783 |
| H | 1.782871 | -2.833301 | 4.111814 |
| H | -0.418304 | 1.319442 | 3.337270 |
| H | 0.136258 | 2.861600 | 4.016810 |
| H | 2.869538 | 3.822208 | 0.240650 |
| H | 4.632307 | 4.063178 | 0.298348 |
| H | 5.374143 | -0.468989 | -0.683502 |
| H | 6.276896 | -1.570863 | 0.384955 |
| H | -0.719177 | -2.771192 | 4.269256 |
| H | -2.872568 | -1.752025 | 3.455817 |
| Y | 2.489548 | 0.371515 | 1.198461 |
| C | -0.621206 | 0.213694 | -0.063401 |
| C | -1.788720 | 0.737332 | -0.634034 |
| C | 0.693400 | 0.973939 | -1.811973 |
| C | -1.675338 | 1.430221 | -1.852273 |
| C | -0.430380 | 1.534737 | -2.446383 |
| H | 1.674218 | 1.055925 | -2.236179 |
| H | -2.565218 | 1.854409 | -2.295353 |
| H | -0.304129 | 2.053703 | -3.387325 |
| N | 0.606547 | 0.351744 | -0.629704 |
| C | -3.053913 | 0.573838 | 0.051303 |
| C | -3.127114 | -0.163335 | 1.250859 |
| C | -5.345409 | 0.965751 | 0.152183 |
| C | -5.429811 | 0.157132 | 1.321433 |
| C | -6.700271 | -0.189403 | 1.996344 |
| C | -7.790841 | -0.688071 | 1.266989 |
| C | -6.787599 | -0.071321 | 3.389179 |
| C | -8.967040 | -1.031333 | 1.929802 |
| H | -7.686433 | -0.839213 | 0.201289 |
| C | -7.973337 | -0.402780 | 4.046423 |
| H | -5.929580 | 0.289259 | 3.942531 |
| C | -9.066588 | -0.877871 | 3.317111 |
| H | -9.804779 | -1.424843 | 1.366869 |
| H | -8.042898 | -0.293414 | 5.121812 |
| H | -9.986973 | -1.136608 | 3.826578 |
| C | -6.502104 | 1.627619 | -0.489255 |
| C | -6.470471 | 1.778253 | -1.887129 |
| C | -7.597803 | 2.126627 | 0.230222 |
| C | -7.546480 | 2.353351 | -2.561235 |
| H | -5.586126 | 1.448004 | -2.414611 |
| C | -8.661550 | 2.723277 | -0.446252 |
| H | -7.617596 | 2.047941 | 1.307184 |
| C | -8.649468 | 2.822084 | -1.840791 |
| H | -7.520893 | 2.440761 | -3.640435 |
| H | -9.502809 | 3.109131 | 0.116079 |
| H | -9.487561 | 3.270036 | -2.360171 |
| N | -4.161971 | 1.138108 | -0.463143 |
| N | -4.312893 | -0.362499 | 1.862099 |
| N | 4.463416 | -3.267576 | -0.386836 |
| N | 4.396945 | 2.757959 | -1.996298 |
| N | -0.628594 | 3.576333 | 1.553808 |

| | | | |
|---|---|---|---|
| C | -0.722962 | 4.089625 | 0.159807 |
| H | -0.791703 | 3.223351 | -0.496085 |
| C | 4.321563 | 1.868893 | -3.194321 |
| H | 3.277234 | 1.572733 | -3.306546 |
| C | 3.394474 | -4.217251 | -0.810833 |
| H | 2.766168 | -3.705130 | -1.551443 |
| C | 0.527912 | 4.874500 | -0.206189 |
| C | 1.107960 | 5.773551 | 0.696594 |
| C | 1.099199 | 4.708619 | -1.472192 |
| C | 2.246486 | 6.497584 | 0.334743 |
| H | 0.675619 | 5.899266 | 1.682647 |
| C | 2.232192 | 5.438860 | -1.838879 |
| H | 0.662979 | 4.000523 | -2.165233 |
| C | 2.809826 | 6.334650 | -0.934653 |
| H | 2.694487 | 7.184261 | 1.042540 |
| H | 2.663715 | 5.303862 | -2.823385 |
| H | 3.693474 | 6.895423 | -1.213309 |
| C | 5.155355 | 0.610522 | -3.000755 |
| C | 4.564597 | -0.646744 | -3.175622 |
| C | 6.514567 | 0.705819 | -2.677285 |
| C | 5.350646 | -1.797998 | -3.072378 |
| H | 3.492261 | -0.744798 | -3.351635 |
| C | 7.290982 | -0.449648 | -2.560691 |
| H | 6.965502 | 1.679169 | -2.517743 |
| C | 6.712216 | -1.705615 | -2.769861 |
| H | 4.887734 | -2.767653 | -3.212292 |
| H | 8.341158 | -0.370696 | -2.306651 |
| H | 7.315371 | -2.602146 | -2.686488 |
| C | 2.528410 | -4.610452 | 0.374343 |
| C | 3.096168 | -5.166250 | 1.527935 |
| C | 1.140263 | -4.440270 | 0.293992 |
| C | 2.283301 | -5.568553 | 2.590049 |
| H | 4.172336 | -5.284908 | 1.593309 |
| C | 0.331053 | -4.838079 | 1.361644 |
| H | 0.728710 | -3.999651 | -0.610091 |
| C | 0.897373 | -5.404835 | 2.507647 |
| H | 2.728468 | -5.995714 | 3.480812 |
| H | -0.740275 | -4.699078 | 1.306179 |
| H | 0.264178 | -5.706738 | 3.333466 |
| C | 4.065346 | -5.440057 | -1.454877 |
| H | 4.700077 | -5.966363 | -0.733840 |
| H | 4.673400 | -5.134710 | -2.313872 |
| H | 3.290952 | -6.127890 | -1.804088 |
| C | 4.768993 | 2.668176 | -4.426851 |
| H | 4.679331 | 2.036444 | -5.313719 |
| H | 5.813896 | 2.976500 | -4.322629 |
| H | 4.139736 | 3.554438 | -4.559054 |
| C | -1.999851 | 4.931328 | 0.023419 |
| H | -1.967897 | 5.789543 | 0.701866 |
| H | -2.879423 | 4.317292 | 0.244401 |
| H | -2.076414 | 5.299315 | -1.002634 |
| H | 5.434599 | -3.548895 | -0.499050 |
| H | -1.283211 | 3.919762 | 2.253423 |

```
H  4.799370  3.686856 -2.097113
C -4.717148 -1.122177 -3.052257
C -3.327166 -1.302311 -3.153106
C -2.642846 -1.943434 -2.117091
C -3.339510 -2.491395 -1.029804
C -4.725040 -2.311520 -0.924735
C -5.394823 -1.573534 -1.911886
C -2.604636 -0.892153 -4.424553
H -2.853386  0.147691 -4.669937
H -2.987044 -1.510899 -5.248364
C -5.483278 -0.524162 -4.215785
H -5.205577  0.520179 -4.401141
H -6.557811 -0.575438 -4.032984
H -5.268033 -1.084483 -5.133111
C -5.481396 -2.971718  0.206406
H -6.551810 -2.816095  0.088915
H -5.172984 -2.559870  1.173280
H -5.276744 -4.048862  0.218028
C -2.585197 -3.324376 -0.020593
H -2.781083 -4.392598 -0.186201
H -2.883476 -3.085396  1.001901
H -1.517105 -3.145380 -0.132090
C -1.073895 -1.034937 -4.331021
C -0.644312 -2.226645 -3.493405
O -1.254204 -2.100422 -2.124970
C  0.882079 -2.190446 -3.181281
C -1.048760 -3.578843 -4.067787
O -6.784825 -1.353028 -1.761868
H -6.984855 -0.395921 -1.961542
O  1.355938 -3.263176 -2.674835
O  1.500483 -1.103449 -3.438310
H -0.645309 -3.686618 -5.080038
H -2.137523 -3.677745 -4.106725
H -0.620602 -4.351943 -3.429060
H -0.643759 -0.161526 -3.854646
H -0.634314 -1.124594 -5.328861
```

(*SSS*)-Δ-[Y.**L**.*R*-Trolox]$^{2+}$

184 atoms

B3LYP/3-21G*//IEFPCM-water//GD3BJ

NIMAG=0

Electronic Energy -7486.878923 a.u.

Gibbs Free Energy -7485.434410 a.u.

| | | | |
|---|---|---|---|
| O | 1.073710 | 2.092708 | 1.269659 |
| O | 3.334217 | 1.438491 | -0.592154 |
| O | 3.194936 | -1.546572 | -0.018789 |
| N | 3.117914 | -1.492986 | 2.972067 |
| N | 1.753267 | 1.046116 | 3.607901 |
| N | 3.782881 | 2.503579 | 1.898026 |
| N | 5.102268 | -0.046541 | 1.265062 |
| N | 0.671371 | -1.192795 | 1.651617 |
| C | 3.025824 | -0.944541 | 4.378118 |
| C | 1.771914 | -0.108439 | 4.580038 |
| C | 2.694747 | 2.119838 | 4.122346 |
| C | 3.086354 | 3.153536 | 3.072268 |
| C | 5.218378 | 2.229854 | 2.262679 |
| C | 5.860026 | 1.260063 | 1.284602 |
| C | 5.493833 | -0.865885 | 2.475265 |
| C | 4.523046 | -2.009564 | 2.748623 |
| C | 2.158300 | -2.652993 | 2.828794 |
| C | 0.363149 | 1.599647 | 3.513736 |
| C | 0.218179 | 2.308800 | 2.185936 |
| C | 3.721114 | 3.426621 | 0.715691 |
| C | 3.779212 | 2.635655 | -0.570511 |
| C | 5.469369 | -0.783179 | 0.011431 |
| C | 4.416091 | -1.819919 | -0.280634 |
| C | 0.757996 | -2.186533 | 2.550177 |
| C | -0.397360 | -2.772602 | 3.095242 |
| C | -1.645201 | -2.285542 | 2.727644 |
| C | -1.737563 | -1.273903 | 1.752895 |
| C | -0.540792 | -0.798591 | 1.192049 |
| H | 3.032276 | -1.773911 | 5.096400 |
| H | 3.914311 | -0.341966 | 4.563699 |
| H | 1.734701 | 0.255963 | 5.613929 |
| H | 0.883203 | -0.711446 | 4.402177 |
| H | 2.228522 | 2.630107 | 4.974724 |
| H | 3.588568 | 1.618951 | 4.491886 |
| H | 3.748082 | 3.892702 | 3.541035 |
| H | 2.216881 | 3.685147 | 2.695639 |
| H | 5.787150 | 3.167884 | 2.283731 |
| H | 5.233103 | 1.808084 | 3.267502 |
| H | 6.906410 | 1.089485 | 1.565394 |
| H | 5.851607 | 1.666826 | 0.273234 |
| H | 6.502701 | -1.272871 | 2.331278 |
| H | 5.527369 | -0.194819 | 3.332953 |
| H | 4.866463 | -2.564223 | 3.630919 |
| H | 4.495119 | -2.708748 | 1.915926 |
| H | 2.476333 | -3.218806 | 1.952227 |
| H | 2.200861 | -3.305533 | 3.705476 |
| H | -0.348159 | 0.769310 | 3.535905 |
| H | 0.132704 | 2.269645 | 4.349935 |
| H | 2.739750 | 3.905146 | 0.719264 |
| H | 4.492140 | 4.202063 | 0.761766 |
| H | 5.439803 | -0.072311 | -0.817048 |
| H | 6.474716 | -1.209880 | 0.077757 |
| H | -0.300761 | -3.578874 | 3.808741 |

| | | | |
|---|---|---|---|
| H | -2.556897 | -2.675620 | 3.159625 |
| Y | 2.565508 | 0.297497 | 1.212000 |
| C | -0.551862 | 0.075350 | 0.046794 |
| C | -1.762310 | 0.588815 | -0.425064 |
| C | 0.644303 | 1.032590 | -1.688270 |
| C | -1.741685 | 1.365324 | -1.600898 |
| C | -0.541328 | 1.535836 | -2.258220 |
| H | 1.602440 | 1.233937 | -2.123600 |
| H | -2.671667 | 1.761923 | -1.978736 |
| H | -0.490007 | 2.064835 | -3.198574 |
| N | 0.648186 | 0.339281 | -0.544400 |
| C | -2.981454 | 0.288705 | 0.292121 |
| C | -2.988795 | -0.695267 | 1.305265 |
| C | -5.260635 | 0.634940 | 0.610690 |
| C | -5.300981 | -0.491785 | 1.486565 |
| C | -6.549514 | -1.090043 | 2.002711 |
| C | -6.599431 | -1.534355 | 3.330644 |
| C | -7.656234 | -1.281565 | 1.159450 |
| C | -7.762148 | -2.123121 | 3.828266 |
| H | -5.728043 | -1.406424 | 3.960053 |
| C | -8.809496 | -1.882757 | 1.658237 |
| H | -7.586820 | -0.995461 | 0.118415 |
| C | -8.870754 | -2.293737 | 2.994473 |
| H | -7.802370 | -2.450066 | 4.860104 |
| H | -9.658736 | -2.038657 | 1.004086 |
| H | -9.773310 | -2.752809 | 3.379457 |
| C | -6.417589 | 1.505950 | 0.314742 |
| C | -6.439685 | 2.169962 | -0.924756 |
| C | -7.457079 | 1.727891 | 1.230322 |
| C | -7.515567 | 2.986104 | -1.270403 |
| H | -5.597792 | 2.033315 | -1.589554 |
| C | -8.520726 | 2.562401 | 0.888865 |
| H | -7.434009 | 1.250296 | 2.198785 |
| C | -8.563705 | 3.179134 | -0.364975 |
| H | -7.533296 | 3.472037 | -2.238072 |
| H | -9.318381 | 2.729640 | 1.601953 |
| H | -9.401472 | 3.812097 | -0.629936 |
| N | -4.109917 | 0.962152 | -0.000425 |
| N | -4.155153 | -1.092361 | 1.855080 |
| N | 4.798454 | -2.965044 | -0.823428 |
| N | 4.214047 | 3.221306 | -1.677376 |
| N | -0.814136 | 3.116192 | 1.977089 |
| C | -0.988005 | 3.755817 | 0.645028 |
| H | -0.960186 | 2.959332 | -0.094125 |
| C | 4.044874 | 2.570422 | -3.013900 |
| H | 2.973671 | 2.399266 | -3.145716 |
| C | 3.814359 | -3.957539 | -1.334672 |
| H | 3.094928 | -3.412414 | -1.957068 |
| C | 0.163699 | 4.705557 | 0.356833 |
| C | 0.664726 | 5.564811 | 1.341152 |
| C | 0.738109 | 4.712152 | -0.918870 |
| C | 1.728517 | 6.422576 | 1.049497 |
| H | 0.233911 | 5.552933 | 2.335781 |

| | | | |
|---|---|---|---|
| C | 1.795275 | 5.575354 | -1.214161 |
| H | 0.366647 | 4.029497 | -1.673208 |
| C | 2.294451 | 6.432575 | -0.229225 |
| H | 2.117540 | 7.077925 | 1.819285 |
| H | 2.231521 | 5.570967 | -2.205549 |
| H | 3.121338 | 7.095466 | -0.452516 |
| C | 4.754890 | 1.225848 | -3.083847 |
| C | 4.016653 | 0.067465 | -3.349201 |
| C | 6.140045 | 1.137144 | -2.904826 |
| C | 4.659932 | -1.165333 | -3.460611 |
| H | 2.933753 | 0.072145 | -3.456008 |
| C | 6.785755 | -0.097406 | -3.030613 |
| H | 6.715178 | 2.026303 | -2.670641 |
| C | 6.047518 | -1.251008 | -3.314694 |
| H | 4.042150 | -2.041287 | -3.625708 |
| H | 7.859177 | -0.158992 | -2.896168 |
| H | 6.551320 | -2.206169 | -3.409105 |
| C | 3.057649 | -4.597280 | -0.184939 |
| C | 3.708293 | -5.194228 | 0.900584 |
| C | 1.661982 | -4.593124 | -0.242350 |
| C | 2.959209 | -5.791320 | 1.918574 |
| H | 4.791574 | -5.188555 | 0.952350 |
| C | 0.912304 | -5.175562 | 0.780167 |
| H | 1.209226 | -4.111485 | -1.099905 |
| C | 1.561244 | -5.779981 | 1.862436 |
| H | 3.463035 | -6.250472 | 2.760803 |
| H | -0.170521 | -5.149065 | 0.739350 |
| H | 0.983469 | -6.230225 | 2.660767 |
| C | 4.567451 | -4.998968 | -2.175512 |
| H | 5.296734 | -5.540094 | -1.562911 |
| H | 5.085367 | -4.515264 | -3.010828 |
| H | 3.850319 | -5.718078 | -2.579472 |
| C | 4.542482 | 3.545265 | -4.091884 |
| H | 5.610353 | 3.744407 | -3.961825 |
| H | 3.990097 | 4.489540 | -4.042687 |
| H | 4.387199 | 3.098642 | -5.076665 |
| C | -2.365301 | 4.432136 | 0.584437 |
| H | -2.463562 | 5.184927 | 1.372796 |
| H | -3.153008 | 3.677110 | 0.685378 |
| H | -2.476377 | 4.924421 | -0.384969 |
| H | 5.787210 | -3.132829 | -0.992246 |
| H | -1.495515 | 3.299724 | 2.710385 |
| H | 4.590706 | 4.165882 | -1.644366 |
| C | -4.803516 | -1.832099 | -1.856650 |
| C | -3.446477 | -1.938848 | -2.201270 |
| C | -2.889013 | -1.051806 | -3.129142 |
| C | -3.681695 | -0.058004 | -3.727755 |
| C | -5.031919 | 0.058683 | -3.374269 |
| C | -5.577248 | -0.809317 | -2.414092 |
| C | -2.591069 | -3.017699 | -1.570477 |
| C | -5.418032 | -2.849481 | -0.919425 |
| H | -4.989382 | -2.774885 | 0.085357 |
| H | -5.229242 | -3.865062 | -1.286797 |

| | | | |
|---|---|---|---|
| H | -6.492253 | -2.694584 | -0.847341 |
| C | -5.906838 | 1.070352 | -4.087192 |
| H | -6.952831 | 0.936149 | -3.806713 |
| H | -5.824822 | 0.940070 | -5.172221 |
| H | -5.609465 | 2.101445 | -3.860861 |
| C | -3.062887 | 0.861201 | -4.757920 |
| H | -2.007257 | 0.614945 | -4.866649 |
| H | -3.157976 | 1.912329 | -4.457689 |
| H | -3.559762 | 0.752995 | -5.730080 |
| C | -1.092763 | -2.732079 | -1.749707 |
| C | -0.755331 | -2.272972 | -3.170984 |
| O | -1.551671 | -1.079436 | -3.510143 |
| C | -1.018989 | -3.371048 | -4.206762 |
| H | -0.306544 | -4.177271 | -4.022099 |
| H | -0.857645 | -2.967455 | -5.211164 |
| H | -2.043143 | -3.749294 | -4.139358 |
| C | 0.743224 | -1.858885 | -3.252150 |
| O | -6.931782 | -0.689346 | -2.017119 |
| H | -7.114442 | 0.263116 | -1.780484 |
| O | 1.037398 | -0.676166 | -3.611190 |
| O | 1.543887 | -2.817031 | -2.951577 |
| H | -2.845104 | -3.993426 | -2.005801 |
| H | -2.812041 | -3.079852 | -0.499306 |
| H | -0.788934 | -1.947387 | -1.059001 |
| H | -0.515573 | -3.624597 | -1.526779 |
